# Temporal trends of no moderate to vigorous physical activity in adolescents: a 16-year trend analysis of 115,926 participants

**DOI:** 10.1186/s12966-025-01862-0

**Published:** 2025-12-04

**Authors:** Sitong Chen, Denver Brown, Christopher D. Pfledderer, Wendy Y. Huang, Mark S. Tremblay

**Affiliations:** 1 Institute for Health and Sport, Victoria University, Melbourne, VIC Australia; 2 Department of Kinesiology, College of Health and Human Sciences, Kansas State University, Manhattan, KS USA; 3 Department of Health Promotion and Behavioral Sciences, School of Public Health, University of Texas Health Science Centre Houston, Austin, TX USA; 4 Department of Sport, Physical Education and Health, Hong Kong Baptist University, Hong Kong, China; 5 Dr Stephen Hui Research Centre for Physical Recreation and Wellness, Hong Kong Baptist University, Hong Kong, China; 6 Children’s Hospital of Eastern Ontario Research Institute, Ottawa, Canada; 7 Department of Pediatrics, University of Ottawa, Ottawa, Canada

**Keywords:** Time-use epidemiology, Physical activity, Trend, School-aged students

## Abstract

**Background:**

Engaging in no moderate-to-vigorous physical activity (MVPA) has been recognized as an important indicator in physical activity (PA) surveillance, as any engagement in MVPA confers health benefits compared to none. Studying the prevalence of no MVPA can provide valuable insights into physical inactivity patterns and inform public health intervention efforts. While some cross-sectional studies have examined this issue, no research has analysed year-to-year trends. Therefore, the aim of this study was to assess trends of no MVPA among adolescents and key subgroups using a nationally representative US sample.

**Methods:**

Data from 2005 to 2021 cycles of the Youth Risk Behavior Surveillance System were used, with 115,926 US adolescents aged 14–17 years included (female: unweighted sample size = 58,582, 50.5%; weighted%=49.4%). Participants self-reported their demographic (sex, age, race/ethnicity, body mass index) and behavioural information (days of ≥ 60 min of MVPA over the past week, and recreational screen time). No MVPA was operationalized as reporting 0 days of ≥ 60 min of MVPA. Trend analysis was performed to assess temporal variations from 2005 to 2021 using a series of binary logistic regression models after controlling for demographic and screen time related variables.

**Results:**

Declining trends in no MVPA were observed among adolescents from 2005 (weighted: 24.3%) to 2021 (weighted: 15.5%). After stratifying by sex, age, race/ethnicity, body mass index and recreational screen time, similar downward trends were shown across all adolescent subgroups consistently (*p* for trend < 0.001). Girls, older adolescents, those who identified as non-White, adolescents with excess weight, and those engaging in more than 2 h of recreational screen time per day tended to report no MVPA at higher rates (all *p* < 0.001) compared to their counterparts.

**Conclusions:**

No MVPA has declined among the US adolescents, especially after 2009. Notably, sociodemographic disparities were observed in no MVPA among different population subgroups. PA promotion strategies targeting girls and older adolescents should be prioritized to further reduce the prevalence of no MVPA.

**Supplementary Information:**

The online version contains supplementary material available at 10.1186/s12966-025-01862-0.

## Introduction

Health benefits of sufficient physical activity (PA) for adolescents have been well documented [1, 2]. Synthesised evidence from review studies demonstrate that engaging in sufficient PA is favourably associated with indicators of health, such as increased fitness levels [3], improved cognitive development [4], lower risk of mental health problems [5] and effective prevention of excess weight [6]. With this convincing evidence base, the 2020 World Health Organisation PA and sedentary behaviour guidelines recommend that adolescents should accumulate an average of 60 min of moderate-to-vigorous PA (MVPA) per day on average across a week [7]. Similar but slightly different from the WHO guidelines [7], the Canadian 24-Hour Movement Guidelines recommend that adolescents should engage in at least 60 min of MVPA per day [8, 9]. Regardless of the specific quantitative recommendations, survey data demonstrate a concerningly low level of MVPA among adolescents [10, 11], which must be addressed as a public health priority [12].

Given the importance of MVPA for health and development, and the need to increase levels of MVPA among adolescents, the surveillance of population levels of MVPA is a necessary part of a public health response [11, 13]. Current PA surveillance primarily focuses on tracking levels of sufficient PA (e.g., prevalence meeting PA guidelines) [14]. International and national surveillance systems, such as the Global School-based Student Health Survey (WHO), the Canadian Health Measures Survey (Statistics Canada) [15], and the Youth Risk Behavior Surveillance System (US Centres for Disease Control and Prevention) [16], provide extensive data for researchers and practitioners to investigate the cross-sectional levels and examine temporal variation of PA in targeted populations. Results from such surveillance efforts can further assist in policy development and implementation to address the “inactivity pandemic” [17], particularly as it relates to addressing existing health-enhancing PA disparities. Among the three surveillance systems, the YRBSS is the most comprehensive, nationally representative, and consistent dataset with repeated measures of PA over time. Additionally, the large sample size and standardized methodology of YRBSS enable robust trend analyses.

In addition to the traditional assessment of the population prevalence of meeting PA guidelines or “sufficient PA”, an alternate approach recently emerging has been to assess the prevalence of “no or low MVPA” [14]. As a relatively new approach, it has been operationalised as less than 1 day per week of 60 min per day of MVPA [14]. However, this term should be defined based on the specific measures used for PA. For example, “no or low MVPA” can be clarified to “no MVPA” if the threshold examined is reporting 0 days of 60 min of MVPA in the past week (responses: 0–7 days). Hereafter, this definition will be referred to as “no MVPA.” In the current literature, “no MVPA” is becoming increasingly important [14], because understanding no MVPA would be beneficial to address the “inactivity pandemic” comprehensively, not only because of needs to promote sufficient PA but also demands to prevent no MVPA, particularly among key subgroups who may be most vulnerable to engaging in no MVPA. Further, as the WHO guidelines promote that “every move counts” towards better health [7], reducing or eliminating “no MVPA” is very important for overall health among young people during a critical developmental stage that is known to predict future MVPA in adulthood [18].

Some emerging data have been published regarding the prevalence of no MVPA. At the global level, approximately 20% of adolescents reported no MVPA [19]. Based on data from the Global School-based Student Health Survey from Latin American countries, the prevalence of no MVPA ranged between 17.2% and 40.4% [14]. Moreover, significant differences related to sex [19], age [19], and region [14, 19] have been observed. These findings can expand our understanding of no MVPA patterns, but more research seeking to identify additional correlates (e.g., race) of no MVPA in adolescents is required to inform intervention priorities given limited public health resources. Furthermore, to date, despite some cross-sectional data [14, 19], no trend analyses on adolescents’ no MVPA have been published. Thus, a major knowledge gap exists regarding temporal trends of no MVPA. Therefore, using repeated cross-sectional data from adolescents living in the United States (US), the aims of this study were to: (1) investigate the prevalence of no MVPA; (2) examine differences in the prevalence of no MVPA across sociodemographic and behavioural subgroups and (3) assess temporal trends of no MVPA.

## Methods

### Study design and participants

This study used data from nine cycles of the Youth Risk Behaviour Surveillance System (YRBSS; 2005, 2007, 2009, 2011, 2013, 2015, 2017, 2019 and 2021). The YRBSS is a biennial, cross-sectional school-based survey of health risk behaviours among a nationally representative sample of high school students residing in the US. The YRBSS uses a three-stage cluster sampling design to recruit students attending public and private schools in grades 9 to 12 (age range: 12 years or younger, 13, 14, 15, 16, 17, and 18 years or older) [16]. The survey was administered in person by trained research staff and completed by students during school hours.

The initial sample for this study consisted of 134,674 participants, of whom participants aged 12 or below (0.3%; small sample size), 13 years (0.1%; small sample size), and 18 years or older (13.9%; out of targeted age range) were excluded. Participants aged 12 years or below and 13 years were excluded from the analyses due to their very small sample sizes, which could lead to unstable estimates and limit the statistical power and reliability of subgroup analyses. A total of 115,926 study respondents (14–17 years) were included in the final analysis. The YRBSS had a 60% response rate per cycle. Data were weighted to be nationally representative according to the complex sampling design and weighting. The CDC’s Institutional Review Board approved the survey. Participants’ legal guardians provided written informed consent. More details about the YRBSS can be found by accessing the study protocol [16].

### Measures

#### Main outcome (days of MVPA)

Participants responded to one survey question that asked: “During the past 7 days, on how many days were you physically active for a total of at least 60 minutes per day”. Response options were 0–7 days. This measure has been validated in the study population and was consistent across all the surveys included in this study [16]. Responses were dichotomized to represent whether participants reported 0 days or not [14].

#### Other variables (demographics and recreational screen time [ST])

Participants provided demographic information pertaining to their sex, age and race/ethnicity, and their information was classified into sex (female and male), age (14-, 15-, 16-and 17-year-old) and race/ethnicity (White, Black or African American, Hispanic/Latino, and All other races). Participants self-reported height and weight from which body mass index (BMI) was calculated and converted to percentiles using the CDC BMI growth charts [20]. Excess weight (i.e., overweight and obesity) was determined using the age- and sex-specific ≥ 85th and ≥ 95th percentiles of BMI, respectively. Participants also responded to two items that asked: “On an average school day, how many hours do you 1) watch TV, and 2) play video or computer games or use a computer for something that is not schoolwork? (Included activities such as Nintendo, Game Boy, PlayStation, Xbox, computer games, and the Internet)”. Response options included “I do not watch TV/play video or computer games or use a computer for something that is not schoolwork on an average school day”, “Less than 1 hour per day”, “1 hour per day”, “2 hours per day”, “3 hours per day”, “4 hours per day” and “5 or more hours per day”. For both items, “less than 1 hour per day” was set as 0.5 h and “5 or more hours per day” as 5 h, according to the previous published studies [21, 22]. This allowed us to sum the ST hours to create a single item representing the total amount of recreational ST. Responses were dichotomized to represent whether participants met the recreational ST recommendation of engaging in ≤ 2 h of ST per day or not [23].

### Statistical analysis

All the variables included in this study were treated as categorical. Missing values for the study variables of interest ranged greatly (missingness %: sex: 0.5%; race/ethnicity: 2.3%; body weight: 8.8%; ST: 5.2%; MVPA: 2.6%). To address potential biases from missing data, we implemented multiple imputations by chained equations [24]. We selected 20 imputations on the basis of the general rule that the number should be at least as large as the fraction of missing information [24]. The imputed descriptive statistic values closely matched the original observed values without significant differences in all the studied variables. Using Taylor linearization, weighted prevalence estimates with 95% confidence intervals (CIs) were calculated to account for complex sampling, producing nationally representative estimates of each variable for each survey year and the combined years. To examine trends in no MVPA prevalence from 2005 to 2021, logistic regression models with time-trend variables were used to examine linear trends across all survey cycles. Sensitivity analyse were also conducted to examine the trends excluding 2021 survey due to the consideration of COVID-19 related impacts. We additionally examined interactions between time and the following factors, including sex, age, race/ethnicity, body weight and recreational ST, to determine whether group disparities widened or narrowed over time. Additional logistic regression models were used to explore associations between no MVPA and selected variables (sex, age, race/ethnicity, body weight, and recreational ST). Adjusted odds ratios (OR) with 95% CIs were provided for all models, controlling for sex, age, race/ethnicity, body weight, and recreational ST. To quantify the magnitude of the trend, we estimated the absolute percentage-point change per cycle with 95% CI using the margins. Analyses used SVY procedures in Stata/IC 18.0 BE (Stata Corp LLC), with statistical significance defined as a 2-tailed p-value < 0.05.

## Results

Of the adolescents included in this study, 49.4% were female (weighted; 95%CI: 48.7–50.0) (Table 1). Fewer 14-year-olds than 15-, 16- and 17-year-olds were included. White adolescents (weighted %: 55.4; 95%CI: 53.3–57.5) were predominant, followed by Hispanic/Latino adolescents. 69.8% (weighted; 95%CI: 69.2–70.4) of adolescents were categorised as normal weight and underweight, while the proportion classified as having overweight and obesity was comparable (weighted: 15.9% [95%CI: 15.5–16.2] vs. 14.3% [95%CI: 13.9–14.8]). Nearly 30% (weighted; 95%CI: 29.7–31.3) of adolescents reported no more than 2 h of recreational ST per day. In terms of days of MVPA, 17.7% (weighted; 95%CI: 17.1–18.3) of adolescents reported no MVPA. Information on sample characteristics by each survey year can be found in Supplementary Table 1.

**Table 1:** Sample characteristics of all participants in this study

Variables	*n* ^a^	% ^a^	% and 95%CI ^b^		
Sex					
Female	58,582	50.5	49.4	48.7	50.0
Male	57,344	49.5	50.6	50.0	51.3
Age					
14 years old	15,880	13.7	14.0	13.4	14.6
15 years old	32,161	27.7	29.1	28.6	29.5
16 years old	34,361	29.6	29.4	29.0	29.9
17 years old	33,524	28.9	27.5	27.0	28.0
Race					
White	52,798	45.5	55.4	53.3	57.5
Black or African American	20,355	17.6	13.8	12.7	14.9
Hispanic/Latino	30,434	26.3	21.3	19.7	22.9
All Other Races	12,339	10.6	9.6	8.8	10.5
Body weight					
Normal and underweight	79,804	68.8	69.8	69.2	70.4
Overweight	18,791	16.2	15.9	15.5	16.2
Obesity	17,331	15.0	14.3	13.9	14.8
Recreational screen time per day					
No more than 2 h	34,416	29.7	30.5	29.7	31.3
More than 2 h	81,510	70.3	69.5	68.7	70.3
Days of moderate to vigorous physical activity per week					
0 days	21,757	18.8	17.7	17.1	18.3
1 day	9,690	8.4	8.0	7.7	8.4
2 days	11,679	10.1	9.8	9.5	10.1
3 days	12,902	11.1	11.1	10.9	11.4
4 days	10,818	9.3	9.5	9.2	9.8
5 days	14,888	12.8	13.2	12.8	13.5
6 days	7,813	6.7	7.1	6.8	7.4
7 days	26,379	22.8	23.6	22.9	24.2

^a^denotes unweighted results, ^b^denotes weighted results

Results regarding the correlates of no MVPA are presented in Table 2. Females were more likely to report no MVPA compared to males (OR = 1.93, 95% CI: 1.83, 2.02, *p* < 0.001). Older adolescents had higher odds (for example, OR _for 17 years_ = 1.55, 95%CI: 1.44, 1.67, *p* < 0.001) of reporting no MVPA compared to the youngest (14 years) age group. Adolescents whom identified as Black or African American (OR = 1.76, 95% CI: 1.62, 1.92, *p* < 0.001), Hispanic/Latino (OR = 1.29, 95% CI: 1.19, 1.39, *p* < 0.001), and other races (OR = 1.31, 95% CI: 1.20, 1.43, *p* < 0.001) showed increased odds of having no MVPA compared to those who identified as White. Adolescents with excess weight exhibited greater odds of no MVPA compared to those of normal or underweight, with obesity (OR = 1.32, 95% CI: 1.25, 1.40, *p* < 0.001) and overweight status (OR = 1.07, 95% CI: 1.01, 1.14, *p* < 0.05) being significant risk factors. In comparison to adolescents engaging in no more than two hours of recreational ST, those engaging in more than two hours had 15% higher odds (OR = 1.15, 95% CI: 1.08, 1.21, *p* < 0.001) of reporting no MVPA.

**Table 2:** Model summary of binary regression for correlates of no moderate to vigorous physical activity days

No moderate to vigorous physical activity days per week	Odds ratio	95%CI		*p*
Sex (reference group = Male)				
Female	1.93	1.83	2.02	0.000
Age (reference group = 14 years)				
15 years old	1.13	1.05	1.21	0.001
16 years old	1.34	1.25	1.44	0.000
17 years old	1.55	1.44	1.67	0.000
Race (reference group = White)				
Black or African American	1.76	1.62	1.92	0.000
Hispanic/Latino	1.29	1.19	1.39	0.000
All Other Races	1.31	1.20	1.43	0.000
Body weight (reference group = Normal and underweight)				
Overweight	1.07	1.01	1.14	0.015
Obesity	1.32	1.25	1.40	0.000
Recreational screen time per day (reference group = No more than 2 h)				
More than 2 h	1.15	1.08	1.21	0.000

*CI* Confidence interval

The prevalence of no MVPA decreased from 24.3% in 2005 to 15.5% in 2021, and the largest drop was between 2009 (22.5%) and 2011 (13.5%). More information on the prevalence of no MVPA by subgroups across various surveys can be found in Table 3.

**Table 3:** Weighted prevalence of no moderate to vigorous physical activity across different survey years (weighted results)

	2005			2007			2009			2011			2013			2015			2017			2019			2021		
	%	95%CI		%	95%CI		%	95%CI		%	95%CI		%	95%CI		%	95%CI		%	95%CI		%	95%CI		%	95%CI	
Overall	24.3	22.8	25.8	24.1	22.5	25.7	22.5	20.8	24.2	13.5	12.5	14.6	14.9	13.5	16.4	13.8	12.4	15.4	15.0	13.3	16.9	16.3	14.6	18.2	15.5	14.3	16.9
Sex																											
Female	30.6	28.5	32.7	30.8	28.3	33.5	29.0	26.9	31.2	17.0	15.8	18.4	19.0	16.9	21.3	16.9	15.0	19.1	19.2	16.8	22.0	19.1	16.9	21.5	19.1	17.3	21.0
Male	18.0	16.5	19.5	17.4	15.9	18.9	16.4	14.6	18.4	10.1	9.0	11.4	10.7	9.5	12.0	10.8	9.2	12.7	10.6	9.6	11.6	13.6	11.9	15.4	12.3	11.1	13.6
Age																											
14 years old	21.1	18.6	23.7	22.2	19.5	25.1	21.3	18.9	23.9	12.0	9.8	14.5	11.4	9.1	14.3	10.3	8.3	12.7	10.7	9.0	12.6	15.5	12.4	19.1	11.8	10.1	13.8
15 years old	23.0	20.6	25.5	21.4	19.4	23.5	22.5	19.8	25.4	12.2	10.7	13.9	12.3	10.7	14.1	12.0	10.2	14.1	13.2	11.7	14.8	13.0	11.2	15.0	14.0	12.4	15.8
16 years old	23.8	22.0	25.6	25.3	22.7	28.1	21.8	19.8	24.0	14.4	13.0	15.9	14.8	13.1	16.7	15.2	13.3	17.3	15.1	12.7	17.9	17.2	15.0	19.7	16.6	15.0	18.5
17 years old	27.7	25.7	29.9	26.6	24.7	28.5	23.7	21.5	26.0	14.7	13.2	16.4	19.0	16.6	21.5	15.9	13.8	18.1	18.8	16.0	21.9	19.1	16.7	21.8	19.1	16.9	21.5
Race																											
White	22.1	20.4	23.8	21.7	19.5	24.1	19.4	17.6	21.4	11.0	9.8	12.4	12.9	11.1	14.9	11.4	9.6	13.6	13.2	10.9	16.0	12.6	11.0	14.3	12.0	10.4	13.8
Black or African American	31.9	28.6	35.3	30.4	27.8	33.2	31.5	28.1	35.0	18.6	16.0	21.4	19.9	18.1	21.8	19.6	16.4	23.2	19.6	17.2	22.2	25.6	21.8	29.7	22.7	19.9	25.8
Hispanic/Latino	25.6	22.5	29.0	25.8	23.4	28.4	23.6	21.7	25.6	15.6	13.9	17.5	15.8	13.5	18.5	15.3	13.5	17.2	15.4	13.4	17.7	18.6	15.6	21.9	18.9	16.7	21.3
All Other Races	24.6	20.7	28.9	25.7	21.9	30.0	25.7	22.1	29.6	16.6	13.8	19.8	17.2	14.5	20.3	15.5	12.4	19.2	17.2	14.5	20.2	17.6	15.1	20.4	16.0	14.1	18.1
Body weight																											
Normal and underweight	23.7	22.2	25.4	23.3	21.4	25.3	21.5	19.7	23.5	13.1	12.0	14.3	14.5	13.1	15.9	13.3	11.7	15.0	14.1	12.3	16.1	15.3	13.6	17.2	14.3	12.9	15.8
Overweight	25.1	22.4	28.0	25.1	22.3	28.1	23.5	20.9	26.3	12.1	10.5	14.0	14.2	12.0	16.8	14.9	12.6	17.4	15.6	13.1	18.5	17.5	14.7	20.8	16.8	14.8	18.9
Obesity	26.1	23.4	28.9	26.7	24.3	29.4	26.5	23.5	29.6	17.3	14.5	20.6	17.9	15.2	20.9	15.3	13.1	17.9	18.6	16.3	21.1	19.2	16.2	22.6	19.5	17.6	21.6
Screen time per day																											
No more than 2 h	21.9	19.9	24.0	21.3	18.9	24.0	19.2	17.1	21.6	11.2	9.6	13.0	12.4	10.1	15.1	12.8	10.3	15.9	14.5	11.8	17.8	16.2	13.4	19.5	15.9	14.2	17.8
More than 2 h	25.4	23.8	27.0	25.3	23.8	27.0	24.1	22.4	25.9	14.6	13.5	15.6	15.9	14.2	17.7	14.3	13.1	15.6	15.2	13.9	16.6	16.3	14.8	18.0	15.4	14.0	17.0

**Fig. 1:**
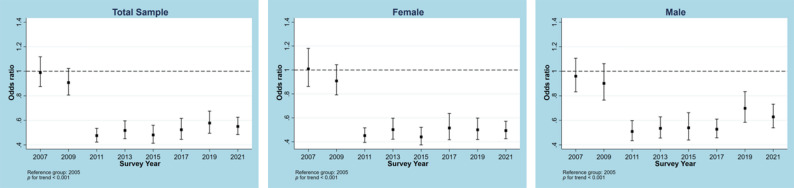
Odds ratio for no moderate to vigorous physical activity across different survey years in the total sample and stratified by sex. The analysis for the total sample was adjusted for sex, age, race, body weight and screen time. The sex-split analyses were adjusted for age, race, body weight and screen time

**Fig. 2:**
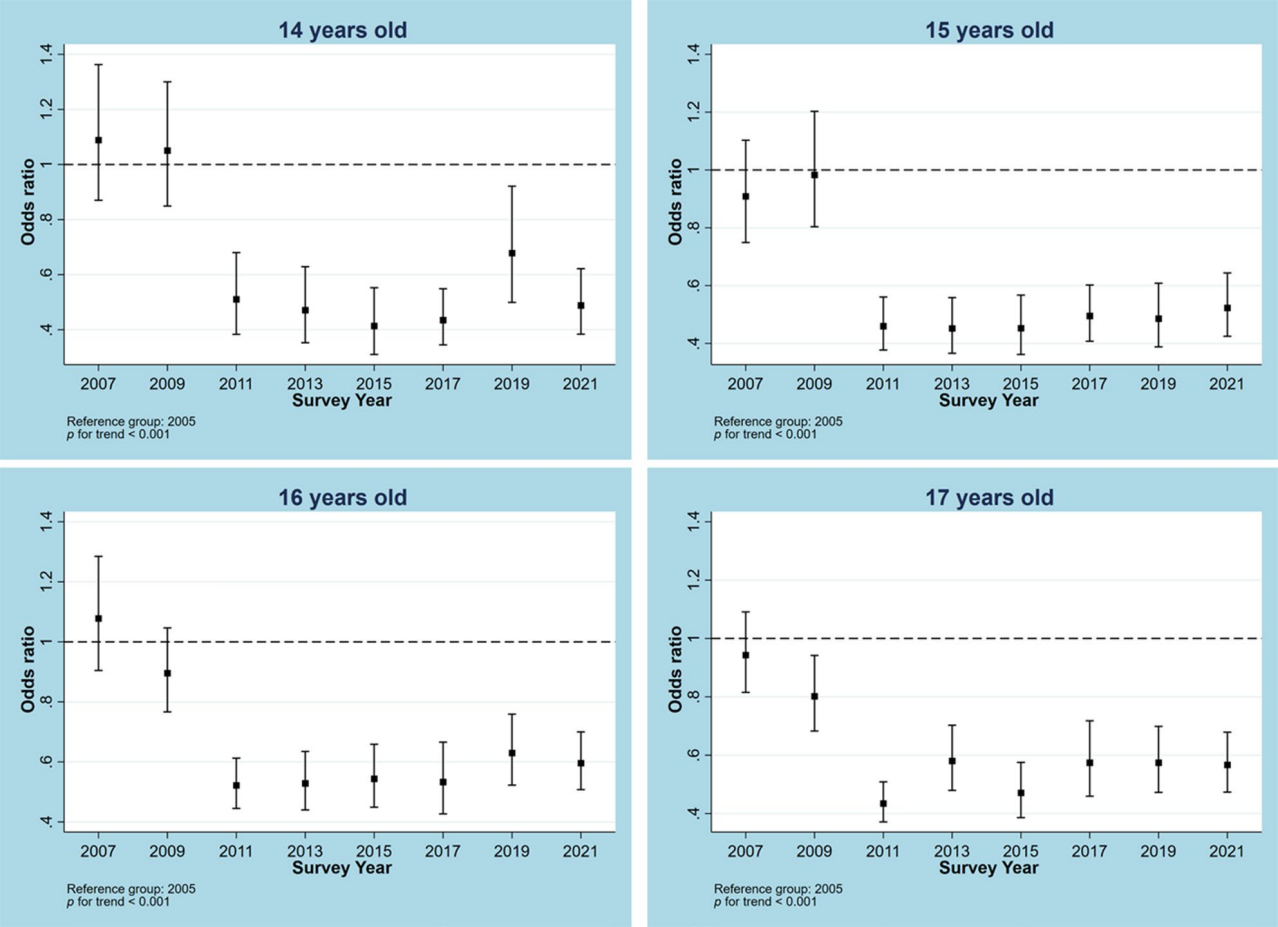
Odds ratio for no moderate to vigorous physical activity across different survey years in the total sample and stratified by age group. The analysis for the total sample was adjusted for sex, race, body weight and screen time. The sex-split analyses were adjusted for sex, race, body weight and screen time

**Fig. 3:**
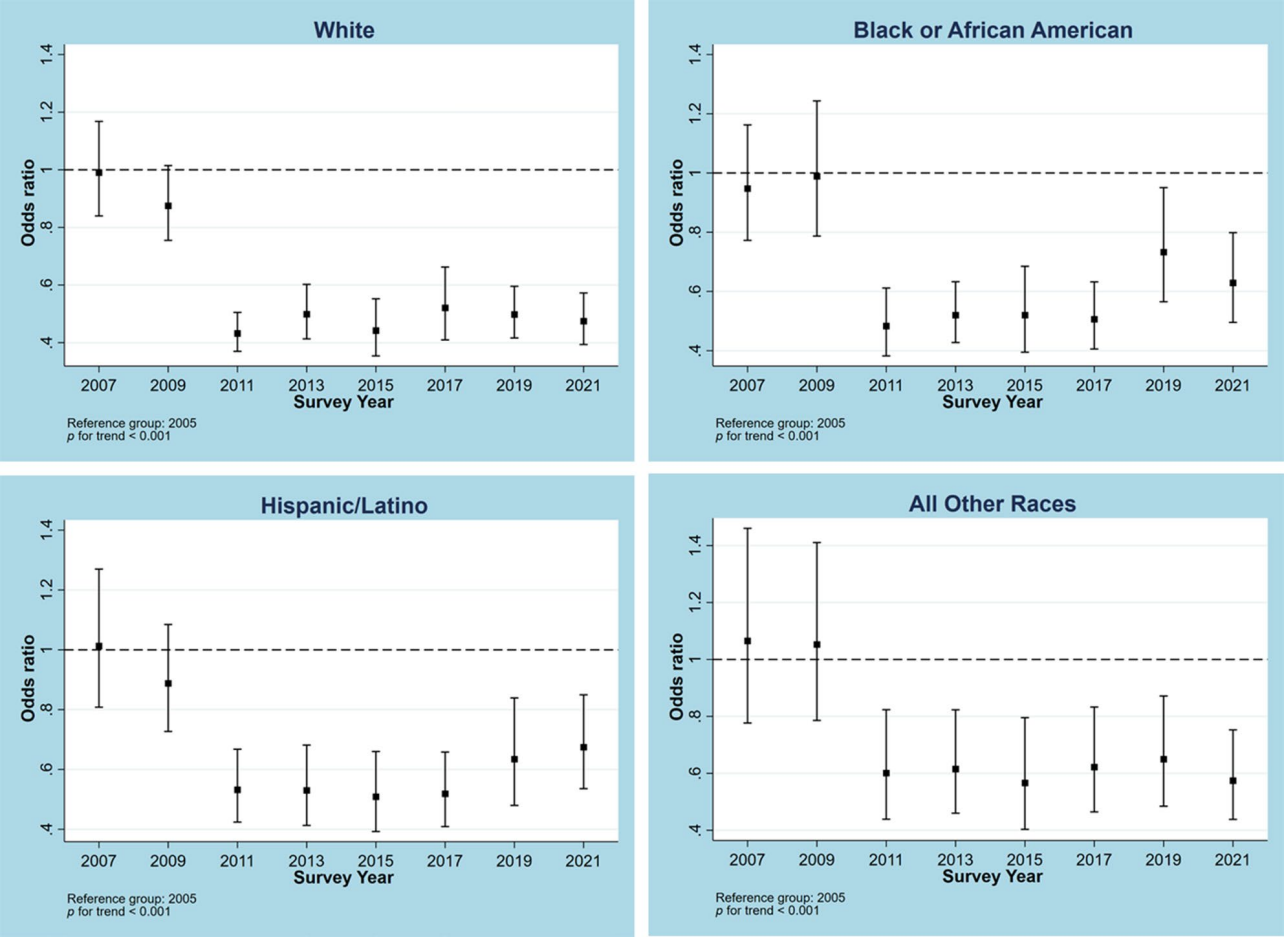
Odds ratio for no moderate to vigorous physical activity across different survey years stratified by race group. The race-split analyses were adjusted for sex, age, body weight and screen time

**Fig. 4:**
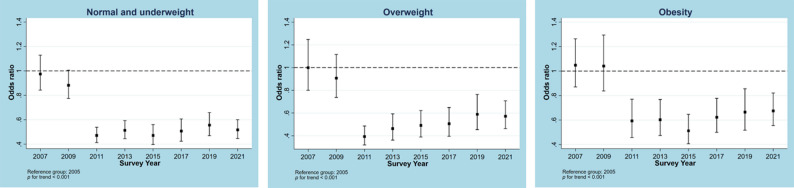
Odds ratio for no moderate to vigorous physical activity across different survey years stratified by body weight. Models controlled for sex, age, race and screen time

**Fig. 5:**
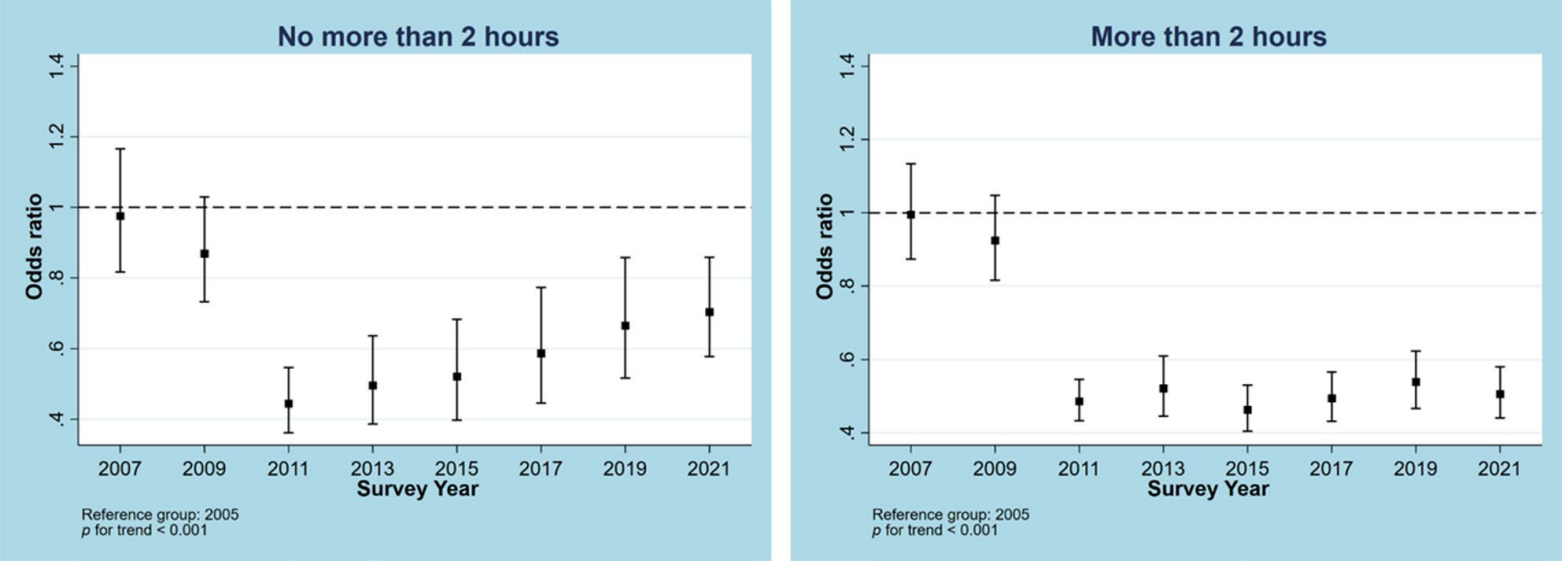
Odds ratio for no moderate to vigorous physical activity across different survey years stratified by screen time. Models controlled for sex, age, race and body weight

Trends for no MVPA from 2005 to 2021 in the overall sample and samples stratified by different characteristics are illustrated in Figs. 1, 2, 3, 4 and 5. Figure 1 shows a general decline (OR: 0.957, 95% CI: 0.950―0.964, p for linear trend < 0.001; change per cycle: −1.15%, 95%CI: −1.39% ― −0.90%; see Supplementary Table 9) in the overall sample and sex-stratified samples (OR for males: 0.949, 95%CI: 0.940―0.957; OR for females: 0.968, 95%CI: 0.959―0.976; both p for linear trend < 0.001), with notable sharp decreases since 2011 and fluctuations observed in males (change per cycle: −0.73%, 95%CI: −0.96% ― −0.49%; see Supplementary Table 9). Figure 2 displays the trends for no MVPA stratified by age groups, indicating similar downward trends in all age groups (all p for trend < 0.001). Figure 3 presents the trends for no MVPA stratified by race/ethnicity, showing significant negative linear trends for adolescents across all race/ethnicity subgroups (all p for trend < 0.001). Figure 4 depicts the trends for no MVPA in adolescents by weight status (normal and underweight, overweight, obesity), showing linearly declining trends across all three groups (all p for trend < 0.001). Figure 5 demonstrates linearly declining trends for no MVPA regardless of recreational ST (both p for trend < 0.001). Across all subgroups, sharp decreases in trends for no MVPA were observed starting from 2011. Results of changes per cycle in other subgroups can be found in Supplementary Table 9. Results of sensitivity analyses are presented in Supplementary Figs. 1–16, revealing the consistent trends with those including 2021 survey. More detailed information on prevalence of days (e.g., at least 1, 2, 3, 4, 5, 6, 7 days) of MVPA across various survey years can be found in Supplementary Tables 2–8.

Significant interactions were found between survey year and several key variables. A survey time × sex interaction (F = 3.00, *p* < 0.005) indicated that declines in no MVPA differed by sex, which reductions were similar between 2007 and 2019, but by 2021 males showed a smaller decrease (β = 0.23, 95% CI: 0.07–0.39, *p* < 0.005), suggesting a widening sex gap. A survey time × age interaction (F (24, 387) = 2.49, *p* < 0.001) showed that declines varied across age groups. Increases were greater in younger adolescents, whereas 17-year-olds group displayed higher prevalence of no MVPA in 2017 (β = 0.28, *p* < 0.05), indicating widening age-related disparities. The survey time × race/ethnicity interaction was not significant (F (24, 387) = 1.02, *p* = 0.43). The survey time × body weight interaction was non-significant (F (16, 395) = 1.00, *p* = 0.45); however, adolescents with obesity had smaller reductions in no MVPA in 2019 (β = 0.16, *p* < 0.05) and 2021 (β = 0.25, *p* < 0.05), indicating widening body weight related gaps. A significant survey time × recreational ST interaction (F (8, 403) = 2.96, *p* < 0.005) showed steeper declines in no MVPA among adolescents with more than 2 h of recreational ST in 2019 (β = −0.21, *p* < 0.05) and 2021 (β = −0.33, *p* = 0.001), indicating narrowing disparities between recreational ST groups. More details can be found in Supplementary Table 10.

## Discussion

### Key findings

This study aimed to investigate the prevalence of no MVPA, correlates of no MVPA, and temporal variation in no MVPA on a biennial basis over a 16-year period (2005 to 2021) among US adolescents. Key findings of this study are: (1) a relatively high prevalence of no MVPA was observed (all combined: 17.7%; the most recent 2021: 15.5%); (2) girls, older adolescents, non-White adolescents, adolescents with excess weight, and adolescents engaging in more than 2 h of recreational ST per day were more likely to report no MVPA; (3) there were significant declines in no MVPA across all adolescents subgroups between 2005 and 2021.

### Interpretations of findings

As recently recommended [14], researchers are encouraged to examine and report “no MVPA” alongside other behaviour related indicators (e.g., sufficient PA, limited ST) in health surveillance systems. In the current study, while we found that nearly one in five adolescents reported no MVPA, it seems impossible to compare our results with the other studies using US samples given the inadequate literature reporting comparable results. In the limited literature, however, we found some data showing the prevalence of no MVPA in adolescents at the global level. For example, our prevalence of no MVPA is lower than the global levels reported by others [19, 25]. Despite this fairly desirable result, enhancing MVPA among adolescents with no MVPA is still crucial from a public health perspective [14] given the health benefits of “every move counts” [7]. Those engaging in no MVPA stand to benefit most given evidence that suggests going from no MVPA to some MVPA may provide the greatest benefits from a relative standpoint [7]. The most recent prevalence of no MVPA in this study (15.5% in 2021) indicates a priority opportunity to target the “least active” adolescents to move more. Given the limited effectiveness of interventions to enhance PA among adolescents with no MVPA [26], more efforts are necessarily needed to explore effective strategies.

Analysis on correlates of no MVPA provides notable insights into where potential intervention efforts may be targeted to have the greatest impact. Our study found that girls were more likely than boys to report no MVPA, consistent with other recent research [14, 19]. This sex inequality has been extensively shown in the literature in relation to other traditional PA indices that consistently reveal that boys are more physically active than girls [10, 27, 28]. Therefore, girls are the intervention priority that not only need to increase MVPA, but also decrease no MVPA, given that sex is a consistent correlate [29–31].

Similar to sex, age is also a consistent correlate of PA [29, 30], as younger adolescents are inclined to be more physically active [10, 28, 32]. These previous studies support our finding that older adolescents have greater odds of reporting no MVPA. The rising prevalence of no MVPA with increasing age could be attributed to limited opportunities and time for different types of sport [33] and physical education [34], while also prioritising other time-use activities [29, 30], such as academic work for high school or university entrance, and peer socialisation.

In our study, adolescents identifying as non-White were more likely to report no MVPA. Race/ethnicity-related health disparities in adolescents are a concerning public health problem in many countries. Evidence has shown that White adolescents are more likely to report higher levels of MVPA in the US [35, 36]. Nevertheless, the present findings may be due to socio-cultural or and environmental barriers, where some ethnic groups have limited access to safe recreational spaces, socio-economic disparities, and/or different cultural norms for PA participation [37]. Addressing these disparities requires culturally tailored interventions and our no MVPA surveillance information highlights this need.

Adolescents with excess weight tended to report higher prevalence of no MVPA, indicating they are at greater risk of an unhealthy PA pattern. This at-risk group needs prioritised attention if implementing PA related interventions, given that physical and psychological barriers make MVPA more difficult for these adolescents [37]. In addition, low self-esteem, body image concerns, and fear of judgment attributed to excess weight could discourage those adolescents from participating in PA. Of note, given the cross-sectional nature of the YRBSS cycles, the observed association between BMI and no MVPA cannot establish directionality and is plausibly bi-directional; moreover, BMI was calculated from self-reported height and weight, which may introduce misclassification and should be considered when interpreting these findings.

Adolescents with more recreational ST had greater odds of reporting no MVPA. This finding is supported by the time-use epidemiology framework that increasing time spent engaging in one behaviour (e.g., recreational ST) takes time away from other behaviours during the course of a 24-hour day (i.e., PA and/or sleep) [38]. Indeed, previous studies have found an inverse relationship between time spent in PA and time spent in SB in children and adolescents [39]. Our finding of a significant association between recreational ST and no MVPA, to some extent, supports the rationale of the Canadian 24-hour movement guidelines’ recommendation of limited recreational ST [9]. For adolescents with coexistence of high recreational ST and no MVPA, this subgroup should be targeted as an intervention priority because of more adverse health effects of combined unhealthy movement behaviours than an isolated approach focused on a single behaviour [40]. Recent evidence has shown that PA interventions could decrease time spent in sedentary behaviour in children and adolescents, and this strategy can be used for this subgroup [41].

Another important finding of our research was the declining temporal trend in the prevalence of no MVPA among US adolescents from 2005 to 2021. The consistency of this finding is remarkable with no discernible variations across the various subgroups, demonstrating a homogenous declining pattern of no MVPA. The declining trend in no MVPA is somewhat promising as it reflects that more adolescents would have started to engage in MVPA. These results show that between 2011 and 2021, more US adolescents participated in MVPA at the different levels (different days of MVPA a week). This finding can also be supported by the data that more adolescents reported at least 1 day of MVPA over time (see supplementary materials). Our data (supplementary materials) and one published study both demonstrate a declining trend in sufficient MVPA from 2011 [36]. Of note, when looking closer at the results, we find particularly sharp decreases after 2009, against slight decreases between 2005 and 2009. This sharp change may reflect the effectiveness of public health initiatives and policies aimed at promoting PA in the US. Two public health related events in 2008 could be mentioned to interpret this finding. The first one was the release of the first guidelines of PA for Americans [42], and the second one was the development of the Comprehensive School-based Physical Activity Program (CSPAP) [43]. These two events were designed to increase PA levels in the US population. Despite the decreased levels of sufficient PA in the US adolescents over the past decades, we assume the situation of no MVPA might be improved through enhanced awareness of the importance of PA and guidance on how to participate in PA from the guidelines, in addition to more opportunities for PA participation created by the CSPAP. For example, from the CSPAP, a whole-of-school PA framework was designed to increase children and adolescents PA through school settings [44] and evidence has demonstrated the beneficial effects of intervention on PA [45]. This could help explain the sharp decreases in no MVPA after 2009. While the post-2009 decline may plausibly reflect the potential effect of the 2008 U.S. Physical Activity Guidelines and/or the CSPAP, alternative explanations such as increased awareness leading to social desirability bias — with some adolescents shifting their responses from “0 days” to “1 day” without actual behavioural change — should also be considered. However, it is challenging to attribute the downward trends to the PA guidelines release and the CSPAP. The primary cause of the downward trend still needs to be further explored. Despite the positive changes, continued efforts are essential to sustain this progress to gain potential health benefits and work towards the US Healthy People 2030 goal of increasing the proportion of young people who do enough aerobic physical activity to 30%. To be cautious, although this finding of decreases in no MVPA over time is encouraging, more insightful analysis is needed to interpret the underlying reasons and assess the impacts on health at the population level. Given extremely rare evidence regarding the changes in no MVPA in adolescents, more relevant studies are needed to better understand the changing pattern.

This study found some disparities in temporal trends of no MVPA. Results of survey time and sex interaction indicated that reductions in no MVPA were less pronounced among males than females, particularly in the most recent survey. This widening sex gap may reflect sex-specific changes in no MVPA. The survey time and age interaction indicated that younger adolescents experienced greater increases in no MVPA than older adolescents. Although race/ethnicity and survey time, and body weight and survey time interactions were not significant, some subgroups had occasional disparities in some particular survey years. However, these might be caused by sampling bias and measurement errors. Finally, the significant survey time and recreational ST interaction indicated a narrowing difference between two different recreational ST groups, which may reflect varying awareness of those with different ST patterns.

### Implications for research and practice

Some implications can be drawn from the research findings. The most prioritised need is to lower the prevalence of no MVPA in US adolescents given the associated health risks, of which more efforts should be placed particularly on girls, older adolescents, non-White adolescents, adolescents with excess weight and those engaging in greater amounts of recreational ST. These key population subgroups are suggested as main intervention targets. Future efforts should focus on specific, evidence-informed strategies to decrease the prevalence of no MVPA, such as improving the quality and inclusivity of physical education and sport opportunities for girls, offering flexible, choice-based activity options and active commuting supports for older adolescents, and creating culturally relevant, accessible programmes for non-White youth and those with excess weight. Despite the improving pattern of no MVPA over the past decades in adolescents (drop from 24.3% to 15.5%), more investments are needed to address the relatively high prevalence (15.5% of 2021). Doing so could help with achieving national public health goals related to PA (e.g., Healthy People 2030). Moreover, the interaction patterns reveal uneven trends in no MVPA among different adolescent subgroups, highlighting the importance of equitable and specific approaches to reduce no MVPA. To achieve these, researchers and practitioners need to understand what kind of interventions can effectively reduce the prevalence of no MVPA. Sociodemographic related characteristics and differences in sufficient MVPA have been well discussed [28, 30], whereas the differences in no MVPA are understudied and poorly understood.

### Study strengths and limitations

Some study strengths are worthwhile to mention. First, our study is the first to demonstrate temporal variations for no MVPA among US adolescents based on the most up-to-date nation-wide behavioural surveillance surveys. Second, analyses were performed based on a range of demographic and behavioural parameters (i.e., sex, age, race, body weight and recreational ST), extending our understanding of no MVPA patterns from a population health perspective. Third, this study was conducted using a large nationally representative sample of US adolescents.

Several limitations must be acknowledged. Although the nationally representative samples were used, those not enrolled, home-schooled, and outside of 14–17 years were excluded in the analysis. As the YRBSS is a national surveillance study, a self-reported questionnaire is the most feasible measure, which is subject to participants’ recall bias and social desirability. Additionally, our total sample consisted of different participants measured in each survey cycle, which resulted in multiple cross-sectional estimates. Future research using data from longitudinal cohorts in diverse populations is warranted to understand how patterns of no MVPA develop through adolescence. Although our study sought to reduce estimation bias through including sex, age and race/ethnicity as covariates, other important variables related to PA, such as family characteristics and built environment characteristics should be considered in future research. Third, as the threshold to identify no or low MVPA is not established, the use of the lowest possible amount of MVPA could limit comparisons between different assessment methods [14]. Fourth, ST measures were limited to school days and focused on traditional screen-based activities, potentially underestimating total ST by excluding weekends and emerging behaviours such as smartphone and social media use. Last but not least, the survey period covered the COVID-19 pandemic, and its associated impacts (e.g., social distance, school lock-down) may influence our results; however, these impacts were not quantifiable in our study.

## Conclusion

Overall, nearly 20% of US adolescents reported no days of the recommended PA level. Differences in sex, age group, race/ethnicity, body weight and recreational ST were observed to be associated with reported no days of MVPA. Despite the downwards trends in no MVPA since 2011, there remains 15% of adolescents who reported no MVPA, demanding greater efforts to further reduce the prevalence. These research findings are beneficial to inform which adolescent subgroups should be targeted as key intervention priorities for health promotion. Due to the limited literature reporting the differences in no MVPA, related studies are certainly needed to clarify and identify more sociodemographic-related correlates, determinants, and antecedents of no MVPA, which will be useful to understand and intervene on no MVPA patterns in the population.

## Supplementary Information

Supplementary Material 1.

## Data Availability

The datasets used and/or analysed during the current study are available from the corresponding author on reasonable request.

## Abbreviations

PA: Physical activity
MVPA: Moderate-to-vigorous physical activity
WHO: World Health Organisation
YRBSS: Youth Risk Behavior Surveillance Risk System
US: United States
CDC: Centre for Disease Control
ST: Screen time
BMI: Body mass index
CIs: Confidence intervals
OR: Odds ratio
CSPAP: Comprehensive School-based Physical Activity Program
