# Supplementary material for: Temporal trends of no moderate to vigorous physical activity in adolescents: a 16-year trend analysis of 115,926 participants
Source: Int J Behav Nutr Phys Act. 2025 Dec 4;23:3. doi: 10.1186/s12966-025-01862-0 (PMC12797897; doi:10.1186/s12966-025-01862-0)
Supplement: Supplementary file 1 — Supplementary Material 1. [file 12966_2025_1862_MOESM1_ESM.docx]

**Supplementary Table 1. Sample characteristics of study participations by different survey years.**

|  | **2005 (n = 11617)** | | | | |  | **2007(n = 11842)** | | | | |  | **2009 (n = 13770)** | | | | |
| --- | --- | --- | --- | --- | --- | --- | --- | --- | --- | --- | --- | --- | --- | --- | --- | --- | --- |
| **Variables** | n ^a^ | % ^a^ | % and 95%CI ^b^ | | |  | n ^a^ | % ^a^ | % and 95%CI ^b^ | | |  | n ^a^ | % ^b^ | % and 95%CI ^b^ | | |
| **Sex** |  |  |  |  |  |  |  |  |  |  |  |  |  |  |  |  |  |
| Female | 6106 | 52.6 | 50.0 | 49.0 | 51.0 |  | 5999 | 50.7 | 49.8 | 48.4 | 51.1 |  | 7087 | 51.5 | 48.1 | 45.5 | 50.7 |
| Male | 5511 | 47.4 | 50.0 | 49.0 | 51.0 |  | 5843 | 49.3 | 50.2 | 48.9 | 51.6 |  | 6683 | 48.5 | 51.9 | 49.3 | 54.5 |
| **Age** |  |  |  |  |  |  |  |  |  |  |  |  |  |  |  |  |  |
| 14 years old | 1189 | 10.2 | 12.0 | 11.1 | 13.0 |  | 1367 | 11.5 | 12.9 | 11.9 | 14.1 |  | 1638 | 11.9 | 13.1 | 11.9 | 14.4 |
| 15 years old | 3187 | 27.4 | 30.7 | 29.6 | 31.9 |  | 3258 | 27.5 | 30.2 | 28.9 | 31.6 |  | 3721 | 27.0 | 28.7 | 27.6 | 29.9 |
| 16 years old | 3558 | 30.6 | 30.2 | 28.9 | 31.5 |  | 3626 | 30.6 | 29.9 | 28.6 | 31.1 |  | 4158 | 30.2 | 30.0 | 28.6 | 31.5 |
| 17 years old | 3683 | 31.7 | 27.1 | 25.9 | 28.4 |  | 3591 | 30.3 | 27.0 | 25.7 | 28.3 |  | 4253 | 30.9 | 28.1 | 26.8 | 29.4 |
| **Race** |  |  |  |  |  |  |  |  |  |  |  |  |  |  |  |  |  |
| White | 5258 | 45.3 | 61.7 | 54.4 | 68.5 |  | 5000 | 42.2 | 59.8 | 52.3 | 66.9 |  | 5940 | 43.1 | 58.0 | 50.5 | 65.2 |
| Black or African American | 2803 | 24.1 | 14.8 | 10.9 | 19.8 |  | 2431 | 20.5 | 15.1 | 11.0 | 20.3 |  | 2288 | 16.6 | 14.3 | 10.4 | 19.3 |
| Hispanic/Latino | 2710 | 23.3 | 15.3 | 11.4 | 20.3 |  | 3341 | 28.2 | 17.1 | 13.0 | 22.2 |  | 4120 | 29.9 | 19.2 | 14.8 | 24.5 |
| All Other Races | 846 | 7.3 | 8.2 | 6.6 | 10.3 |  | 1070 | 9.0 | 8.0 | 6.2 | 10.3 |  | 1422 | 10.3 | 8.5 | 5.9 | 12.1 |
| **Body weight** |  |  |  |  |  |  |  |  |  |  |  |  |  |  |  |  |  |
| Normal and underweight | 8019 | 69.0 | 70.0 | 68.5 | 71.5 |  | 8015 | 67.7 | 70.0 | 67.9 | 72.1 |  | 9696 | 70.4 | 71.7 | 70.2 | 73.1 |
| Overweight | 1837 | 15.8 | 15.4 | 14.4 | 16.5 |  | 1991 | 16.8 | 16.3 | 15.2 | 17.4 |  | 2230 | 16.2 | 15.6 | 14.6 | 16.6 |
| Obesity | 1761 | 15.2 | 14.5 | 13.7 | 15.4 |  | 1836 | 15.5 | 13.7 | 12.5 | 15.1 |  | 1844 | 13.4 | 12.8 | 11.8 | 13.8 |
| **Recreational screen time per day** |  |  |  |  |  |  |  |  |  |  |  |  |  |  |  |  |  |
| No more than 2 hours | 3532 | 30.4 | 31.6 | 29.5 | 33.9 |  | 3647 | 30.8 | 32.2 | 29.6 | 35.0 |  | 4393 | 31.9 | 33.3 | 31.4 | 35.3 |
| More than 2 hours | 8085 | 69.6 | 68.4 | 66.1 | 70.5 |  | 8195 | 69.2 | 67.8 | 65.0 | 70.4 |  | 9377 | 68.1 | 66.7 | 64.7 | 68.6 |
| **Days of moderate to vigorous physical activity per week** | | | | | |  |  |  |  |  |  |  |  |  |  |  |  |
| 0 days | 3055 | 26.3 | 24.3 | 22.8 | 25.8 |  | 2993 | 25.3 | 24.1 | 22.5 | 25.7 |  | 3327 | 24.2 | 22.5 | 20.8 | 24.2 |
| 1 day | 1257 | 10.8 | 11.0 | 10.3 | 11.8 |  | 1258 | 10.6 | 10.2 | 9.4 | 11.0 |  | 1404 | 10.2 | 10.0 | 9.2 | 11.0 |
| 2 days | 1220 | 10.5 | 10.5 | 9.6 | 11.5 |  | 1263 | 10.7 | 10.4 | 9.4 | 11.4 |  | 1532 | 11.1 | 10.8 | 10.1 | 11.5 |
| 3 days | 1177 | 10.1 | 10.1 | 9.1 | 11.2 |  | 1294 | 10.9 | 11.4 | 10.7 | 12.2 |  | 1448 | 10.5 | 10.2 | 9.5 | 10.9 |
| 4 days | 882 | 7.6 | 7.5 | 6.9 | 8.3 |  | 971 | 8.2 | 8.1 | 7.3 | 9.1 |  | 1127 | 8.2 | 8.6 | 7.9 | 9.5 |
| 5 days | 1308 | 11.3 | 11.8 | 10.9 | 12.7 |  | 1367 | 11.5 | 12.1 | 11.1 | 13.1 |  | 1620 | 11.8 | 11.9 | 11.1 | 12.6 |
| 6 days | 707 | 6.1 | 6.6 | 5.9 | 7.4 |  | 699 | 5.9 | 6.0 | 5.3 | 6.9 |  | 897 | 6.5 | 7.2 | 6.3 | 8.1 |
| 7 days | 2011 | 17.3 | 18.2 | 17.0 | 19.5 |  | 1997 | 16.9 | 17.7 | 16.6 | 18.8 |  | 2415 | 17.5 | 18.8 | 17.7 | 20.0 |
|  | **2011 (n = 13075)** | | | | |  | **2013 (n = 11219)** | | | | |  | **2015 (n = 13433)** | | | | |
| **Variables** | n ^a^ | % ^a^ | % and 95%CI ^b^ | | |  | n ^a^ | % ^a^ | % and 95%CI ^b^ | | |  | n ^a^ | % ^b^ | % and 95%CI ^b^ | | |
| **Sex** |  |  |  |  |  |  |  |  |  |  |  |  |  |  |  |  |  |
| Female | 6625 | 50.7 | 48.9 | 47.0 | 50.9 |  | 5565 | 49.6 | 50.7 | 49.3 | 52.0 |  | 6762 | 50.3 | 49.2 | 46.3 | 52.1 |
| Male | 6450 | 49.3 | 51.1 | 49.1 | 53.0 |  | 5654 | 50.4 | 49.3 | 48.0 | 50.7 |  | 6671 | 49.7 | 50.8 | 47.9 | 53.7 |
| **Age** |  |  |  |  |  |  |  |  |  |  |  |  |  |  |  |  |  |
| 14 years old | 1569 | 12.0 | 13.5 | 12.0 | 15.2 |  | 1375 | 12.3 | 12.0 | 10.7 | 13.3 |  | 1691 | 12.6 | 11.8 | 10.4 | 13.4 |
| 15 years old | 3486 | 26.7 | 28.7 | 27.1 | 30.3 |  | 3124 | 27.8 | 28.7 | 27.4 | 29.9 |  | 3832 | 28.5 | 30.7 | 29.1 | 32.3 |
| 16 years old | 4085 | 31.2 | 30.2 | 29.1 | 31.4 |  | 3223 | 28.7 | 30.1 | 28.6 | 31.6 |  | 4049 | 30.1 | 29.5 | 28.0 | 31.1 |
| 17 years old | 3935 | 30.1 | 27.6 | 26.0 | 29.3 |  | 3497 | 31.2 | 29.3 | 28.0 | 30.6 |  | 3861 | 28.7 | 28.0 | 26.8 | 29.2 |
| **Race** |  |  |  |  |  |  |  |  |  |  |  |  |  |  |  |  |  |
| White | 5401 | 41.3 | 56.4 | 48.8 | 63.6 |  | 4638 | 41.3 | 55.1 | 47.0 | 63.0 |  | 6107 | 45.5 | 54.0 | 46.2 | 61.6 |
| Black or African American | 2338 | 17.9 | 14.2 | 10.8 | 18.6 |  | 2462 | 21.9 | 14.3 | 10.1 | 19.9 |  | 1472 | 11.0 | 13.9 | 10.5 | 18.2 |
| Hispanic/Latino | 3973 | 30.4 | 20.3 | 15.2 | 26.5 |  | 2875 | 25.6 | 21.4 | 16.6 | 27.2 |  | 4414 | 32.9 | 22.4 | 16.8 | 29.3 |
| All Other Races | 1363 | 10.4 | 9.1 | 7.3 | 11.4 |  | 1244 | 11.1 | 9.2 | 7.5 | 11.2 |  | 1440 | 10.7 | 9.7 | 7.7 | 12.1 |
| **Body weight** |  |  |  |  |  |  |  |  |  |  |  |  |  |  |  |  |  |
| Normal and underweight | 9191 | 70.3 | 71.5 | 69.6 | 73.4 |  | 7788 | 69.4 | 69.9 | 68.0 | 71.7 |  | 9151 | 68.1 | 69.8 | 67.7 | 71.9 |
| Overweight | 2069 | 15.8 | 15.3 | 14.4 | 16.2 |  | 1832 | 16.3 | 16.3 | 15.3 | 17.5 |  | 2248 | 16.7 | 16.0 | 15.0 | 17.1 |
| Obesity | 1815 | 13.9 | 13.2 | 11.8 | 14.7 |  | 1599 | 14.3 | 13.8 | 12.5 | 15.1 |  | 2034 | 15.1 | 14.1 | 12.8 | 15.6 |
| **Recreational screen time per day** |  |  |  |  |  |  |  |  |  |  |  |  |  |  |  |  |  |
| No more than 2 hours | 3782 | 28.9 | 30.5 | 28.8 | 32.2 |  | 2848 | 25.4 | 27.7 | 25.3 | 30.3 |  | 4120 | 30.7 | 31.4 | 29.2 | 33.7 |
| More than 2 hours | 9293 | 71.1 | 69.5 | 67.8 | 71.2 |  | 8371 | 74.6 | 72.3 | 69.7 | 74.7 |  | 9313 | 69.3 | 68.6 | 66.3 | 70.8 |
| **Days of moderate to vigorous physical activity per week** | | | | | |  |  |  |  |  |  |  |  |  |  |  |  |
| 0 days | 1949 | 14.9 | 13.5 | 12.5 | 14.6 |  | 1774 | 15.8 | 14.9 | 13.5 | 16.4 |  | 2007 | 14.9 | 13.8 | 12.4 | 15.4 |
| 1 day | 1021 | 7.8 | 7.1 | 6.5 | 7.8 |  | 806 | 7.2 | 6.7 | 6.1 | 7.4 |  | 989 | 7.4 | 6.6 | 5.8 | 7.4 |
| 2 days | 1267 | 9.7 | 9.0 | 8.2 | 9.8 |  | 1065 | 9.5 | 9.8 | 8.8 | 10.8 |  | 1284 | 9.6 | 8.9 | 8.1 | 9.7 |
| 3 days | 1403 | 10.7 | 10.4 | 9.8 | 11.0 |  | 1220 | 10.9 | 11.4 | 10.6 | 12.2 |  | 1540 | 11.5 | 11.4 | 10.7 | 12.1 |
| 4 days | 1268 | 9.7 | 10.1 | 9.5 | 10.8 |  | 1081 | 9.6 | 9.8 | 8.9 | 10.8 |  | 1325 | 9.9 | 10.0 | 9.3 | 10.8 |
| 5 days | 1665 | 12.7 | 13.1 | 12.2 | 14.0 |  | 1472 | 13.1 | 13.3 | 12.3 | 14.3 |  | 1953 | 14.5 | 15.0 | 14.0 | 16.2 |
| 6 days | 948 | 7.3 | 8.1 | 7.0 | 9.2 |  | 721 | 6.4 | 6.9 | 6.3 | 7.6 |  | 897 | 6.7 | 6.9 | 6.0 | 8.0 |
| 7 days | 3554 | 27.2 | 28.8 | 27.2 | 30.5 |  | 3080 | 27.5 | 27.2 | 25.6 | 28.9 |  | 3438 | 25.6 | 27.4 | 25.5 | 29.3 |
|  | **2017 (n = 12888)** | | | | |  | **2019 (n = 11974)** | | | | |  | **2021 (n = 16108)** | | | | |
| **Variables** | n ^a^ | % ^a^ | % and 95%CI ^b^ | | |  | n ^a^ | % ^a^ | % and 95%CI ^b^ | | |  | n ^a^ | % ^b^ | % and 95%CI ^b^ | | |
| **Sex** |  |  |  |  |  |  |  |  |  |  |  |  |  |  |  |  |  |
| Female | 6629 | 51.4 | 50.9 | 48.4 | 53.4 |  | 6101 | 51.0 | 49.3 | 47.9 | 50.8 |  | 7708 | 47.9 | 48.0 | 46.3 | 49.7 |
| Male | 6259 | 48.6 | 49.1 | 46.6 | 51.6 |  | 5873 | 49.0 | 50.7 | 49.2 | 52.1 |  | 8400 | 52.1 | 52.0 | 50.3 | 53.7 |
| **Age** |  |  |  |  |  |  |  |  |  |  |  |  |  |  |  |  |  |
| 14 years old | 1928 | 15.0 | 13.5 | 12.3 | 14.8 |  | 1711 | 14.3 | 13.8 | 12.7 | 15.1 |  | 3412 | 21.2 | 21.3 | 19.8 | 22.8 |
| 15 years old | 3609 | 28.0 | 29.0 | 27.8 | 30.2 |  | 3491 | 29.2 | 28.8 | 27.5 | 30.1 |  | 4453 | 27.6 | 27.0 | 25.7 | 28.4 |
| 16 years old | 3708 | 28.8 | 29.4 | 28.1 | 30.7 |  | 3651 | 30.5 | 29.8 | 28.7 | 31.0 |  | 4303 | 26.7 | 26.4 | 25.5 | 27.3 |
| 17 years old | 3643 | 28.3 | 28.2 | 26.9 | 29.5 |  | 3121 | 26.1 | 27.5 | 26.3 | 28.7 |  | 3940 | 24.5 | 25.3 | 24.0 | 26.7 |
| **Race** |  |  |  |  |  |  |  |  |  |  |  |  |  |  |  |  |  |
| White | 5708 | 44.3 | 53.6 | 46.8 | 60.2 |  | 6032 | 50.4 | 50.9 | 43.6 | 58.1 |  | 8714 | 54.1 | 50.2 | 42.1 | 58.3 |
| Black or African American | 2455 | 19.0 | 13.3 | 10.4 | 17.0 |  | 1866 | 15.6 | 12.4 | 9.2 | 16.5 |  | 2240 | 13.9 | 12.2 | 9.2 | 16.0 |
| Hispanic/Latino | 3169 | 24.6 | 22.8 | 17.5 | 29.0 |  | 2720 | 22.7 | 26.3 | 20.3 | 33.3 |  | 3112 | 19.3 | 25.4 | 19.2 | 32.8 |
| All Other Races | 1556 | 12.1 | 10.3 | 8.8 | 12.0 |  | 1356 | 11.3 | 10.5 | 7.9 | 13.8 |  | 2042 | 12.7 | 12.2 | 9.3 | 15.8 |
| **Body weight** |  |  |  |  |  |  |  |  |  |  |  |  |  |  |  |  |  |
| Normal and underweight | 8844 | 68.6 | 69.3 | 67.6 | 71.0 |  | 8266 | 69.0 | 68.2 | 65.8 | 70.5 |  | 10834 | 67.3 | 68.0 | 66.1 | 69.7 |
| Overweight | 2107 | 16.3 | 15.6 | 14.7 | 16.5 |  | 1926 | 16.1 | 16.2 | 15.0 | 17.4 |  | 2551 | 15.8 | 16.0 | 15.2 | 16.9 |
| Obesity | 1937 | 15.0 | 15.1 | 13.9 | 16.4 |  | 1782 | 14.9 | 15.6 | 14.2 | 17.2 |  | 2723 | 16.9 | 16.0 | 14.8 | 17.4 |
| **Recreational screen time per day** |  |  |  |  |  |  |  |  |  |  |  |  |  |  |  |  |  |
| No more than 2 hours | 4266 | 33.1 | 33.1 | 30.9 | 35.4 |  | 3931 | 32.8 | 32.2 | 30.6 | 34.0 |  | 3897 | 24.2 | 23.9 | 22.3 | 25.5 |
| More than 2 hours | 8622 | 66.9 | 66.9 | 64.6 | 69.1 |  | 8043 | 67.2 | 67.8 | 66.0 | 69.4 |  | 12211 | 75.8 | 76.1 | 74.5 | 77.7 |
| **Days of moderate to vigorous physical activity per week** | |  |  |  |  |  |  |  |  |  |  |  |  |  |  |  |  |
| 0 days | 2102 | 16.3 | 15.0 | 13.3 | 16.9 |  | 2033 | 17.0 | 16.3 | 14.6 | 18.2 |  | 2517 | 15.6 | 15.5 | 14.3 | 16.9 |
| 1 day | 951 | 7.4 | 7.1 | 5.9 | 8.5 |  | 876 | 7.3 | 6.4 | 5.9 | 7.1 |  | 1128 | 7.0 | 7.1 | 6.3 | 8.0 |
| 2 days | 1282 | 9.9 | 9.4 | 8.5 | 10.4 |  | 1206 | 10.1 | 9.8 | 9.2 | 10.5 |  | 1560 | 9.7 | 9.8 | 9.0 | 10.5 |
| 3 days | 1536 | 11.9 | 11.7 | 10.7 | 12.7 |  | 1477 | 12.3 | 12.0 | 11.3 | 12.6 |  | 1807 | 11.2 | 11.8 | 11.2 | 12.5 |
| 4 days | 1278 | 9.9 | 9.7 | 9.0 | 10.4 |  | 1209 | 10.1 | 10.7 | 9.9 | 11.6 |  | 1677 | 10.4 | 10.5 | 9.7 | 11.3 |
| 5 days | 1737 | 13.5 | 13.9 | 12.7 | 15.2 |  | 1614 | 13.5 | 13.7 | 12.8 | 14.6 |  | 2152 | 13.4 | 13.8 | 13.0 | 14.6 |
| 6 days | 849 | 6.6 | 6.8 | 6.1 | 7.5 |  | 833 | 7.0 | 7.5 | 6.6 | 8.5 |  | 1262 | 7.8 | 7.8 | 7.0 | 8.7 |
| 7 days | 3153 | 24.5 | 26.5 | 24.4 | 28.8 |  | 2726 | 22.8 | 23.6 | 22.3 | 24.9 |  | 4005 | 24.9 | 23.8 | 22.3 | 25.3 |

^a^ denotes unweighted results; ^b^ denotes weighted results.

**Supplementary Table 2. Weighted prevalence of at least one day of moderate to vigorous physical activity per week across different survey years.**

|  | **2005** | | |  | **2007** | | |  | **2009** | | |
| --- | --- | --- | --- | --- | --- | --- | --- | --- | --- | --- | --- |
|  | **%** | **95%CI** | |  | **%** | **95%CI** | |  | **%** | **95%CI** | |
| **Overall** | 64.7 | 62.8 | 66.5 |  | 65.7 | 64.0 | 67.5 |  | 67.5 | 65.4 | 69.5 |
|  |  |  |  |  |  |  |  |  |  |  |  |
| **Sex** |  |  |  |  |  |  |  |  |  |  |  |
| Female | 57.1 | 54.8 | 59.3 |  | 57.5 | 54.9 | 60.1 |  | 59.0 | 56.7 | 61.3 |
| Male | 72.3 | 70.1 | 74.5 |  | 73.9 | 72.1 | 75.6 |  | 75.4 | 72.5 | 78.0 |
|  |  |  |  |  |  |  |  |  |  |  |  |
| **Age** |  |  |  |  |  |  |  |  |  |  |  |
| 14 years old | 67.4 | 64.1 | 70.5 |  | 67.9 | 64.5 | 71.2 |  | 68.9 | 65.8 | 71.7 |
| 15 years old | 65.1 | 62.1 | 68.0 |  | 68.0 | 65.6 | 70.2 |  | 68.4 | 65.5 | 71.1 |
| 16 years old | 65.0 | 62.5 | 67.3 |  | 64.8 | 62.0 | 67.4 |  | 68.3 | 65.8 | 70.7 |
| 17 years old | 62.8 | 60.5 | 65.0 |  | 63.3 | 61.1 | 65.4 |  | 65.1 | 62.4 | 67.7 |
|  |  |  |  |  |  |  |  |  |  |  |  |
| **Race** |  |  |  |  |  |  |  |  |  |  |  |
| White | 66.9 | 64.8 | 68.9 |  | 68.7 | 66.4 | 70.8 |  | 71.0 | 68.4 | 73.4 |
| Black or African American | 58.0 | 54.6 | 61.3 |  | 59.2 | 56.5 | 61.8 |  | 60.6 | 57.0 | 64.1 |
| Hispanic/Latino | 62.5 | 58.9 | 66.0 |  | 61.7 | 58.4 | 64.9 |  | 64.2 | 61.9 | 66.4 |
| All Other Races | 64.5 | 59.6 | 69.1 |  | 64.8 | 59.6 | 69.7 |  | 62.8 | 57.8 | 67.4 |
|  |  |  |  |  |  |  |  |  |  |  |  |
| **Body weight** |  |  |  |  |  |  |  |  |  |  |  |
| Normal and underweight | 65.6 | 63.7 | 67.3 |  | 66.9 | 64.6 | 69.0 |  | 68.9 | 66.7 | 71.1 |
| Overweight | 63.4 | 60.0 | 66.8 |  | 63.9 | 60.9 | 66.9 |  | 66.2 | 63.1 | 69.1 |
| Obesity | 61.9 | 58.7 | 65.0 |  | 62.1 | 59.3 | 64.9 |  | 61.2 | 57.4 | 64.9 |
|  |  |  |  |  |  |  |  |  |  |  |  |
| **Recreational screen time per day** |  |  |  |  |  |  |  |  |  |  |  |
| No more than 2 hours | 68.5 | 65.9 | 70.9 |  | 69.2 | 65.9 | 72.4 |  | 72.2 | 69.1 | 75.2 |
| More than 2 hours | 62.9 | 60.9 | 64.9 |  | 64.1 | 62.3 | 65.8 |  | 65.1 | 63.2 | 67.0 |
|  | **2011** | | |  | **2013** | | |  | **2015** | | |
|  | **%** | **95%CI** | |  | **%** | **95%CI** | |  | **%** | **95%CI** | |
| **Overall** | 79.4 | 77.9 | 80.8 |  | 78.4 | 76.4 | 80.2 |  | 79.6 | 77.4 | 81.7 |
|  |  |  |  |  |  |  |  |  |  |  |  |
| **Sex** |  |  |  |  |  |  |  |  |  |  |  |
| Female | 73.6 | 71.6 | 75.5 |  | 71.9 | 69.2 | 74.6 |  | 74.8 | 71.8 | 77.7 |
| Male | 85.0 | 83.5 | 86.3 |  | 85.0 | 83.4 | 86.4 |  | 84.3 | 81.9 | 86.4 |
|  |  |  |  |  |  |  |  |  |  |  |  |
| **Age** |  |  |  |  |  |  |  |  |  |  |  |
| 14 years old | 80.3 | 77.2 | 83.0 |  | 83.6 | 79.8 | 86.7 |  | 83.0 | 80.0 | 85.6 |
| 15 years old | 81.7 | 79.6 | 83.7 |  | 81.4 | 79.1 | 83.5 |  | 82.2 | 79.4 | 84.6 |
| 16 years old | 78.4 | 76.5 | 80.1 |  | 78.0 | 75.8 | 80.0 |  | 77.8 | 75.1 | 80.3 |
| 17 years old | 77.7 | 75.7 | 79.6 |  | 73.7 | 70.7 | 76.4 |  | 77.4 | 74.3 | 80.2 |
|  |  |  |  |  |  |  |  |  |  |  |  |
| **Race** |  |  |  |  |  |  |  |  |  |  |  |
| White | 82.4 | 80.5 | 84.1 |  | 81.5 | 78.8 | 83.9 |  | 82.6 | 79.6 | 85.3 |
| Black or African American | 74.2 | 71.1 | 77.1 |  | 71.1 | 69.0 | 73.2 |  | 74.0 | 69.4 | 78.1 |
| Hispanic/Latino | 76.3 | 73.9 | 78.4 |  | 76.3 | 73.1 | 79.3 |  | 77.3 | 74.8 | 79.6 |
| All Other Races | 76.0 | 72.3 | 79.4 |  | 75.5 | 72.2 | 78.6 |  | 76.5 | 72.1 | 80.4 |
|  |  |  |  |  |  |  |  |  |  |  |  |
| **Body weight** |  |  |  |  |  |  |  |  |  |  |  |
| Normal and underweight | 80.1 | 78.5 | 81.7 |  | 79.2 | 77.5 | 80.9 |  | 80.7 | 78.3 | 82.9 |
| Overweight | 80.9 | 78.7 | 82.8 |  | 78.6 | 75.4 | 81.6 |  | 78.0 | 74.9 | 80.9 |
| Obesity | 73.7 | 70.0 | 77.1 |  | 73.7 | 69.4 | 77.6 |  | 76.3 | 73.3 | 79.1 |
|  |  |  |  |  |  |  |  |  |  |  |  |
| **Recreational screen time per day** |  |  |  |  |  |  |  |  |  |  |  |
| No more than 2 hours | 83.0 | 80.8 | 85.1 |  | 83.2 | 80.2 | 85.8 |  | 82.0 | 78.0 | 85.4 |
| More than 2 hours | 77.8 | 76.5 | 79.1 |  | 76.5 | 74.3 | 78.6 |  | 78.6 | 76.7 | 80.3 |
|  | **2017** | | |  | **2019** | | |  | **2021** | | |
|  | **%** | **95%CI** | |  | **%** | **95%CI** | |  | **%** | **95%CI** | |
| **Overall** | 77.9 | 74.8 | 80.7 |  | 77.3 | 75.1 | 79.4 |  | 77.4 | 75.5 | 79.1 |
|  |  |  |  |  |  |  |  |  |  |  |  |
| **Sex** |  |  |  |  |  |  |  |  |  |  |  |
| Female | 72.0 | 67.4 | 76.2 |  | 73.0 | 70.2 | 75.6 |  | 72.4 | 70.2 | 74.6 |
| Male | 84.0 | 82.5 | 85.5 |  | 81.5 | 79.2 | 83.5 |  | 81.9 | 80.1 | 83.6 |
|  |  |  |  |  |  |  |  |  |  |  |  |
| **Age** |  |  |  |  |  |  |  |  |  |  |  |
| 14 years old | 82.4 | 79.3 | 85.2 |  | 78.4 | 74.2 | 82.2 |  | 81.5 | 79.0 | 83.7 |
| 15 years old | 80.0 | 76.8 | 82.9 |  | 80.6 | 78.2 | 82.8 |  | 78.4 | 75.4 | 81.0 |
| 16 years old | 77.4 | 73.4 | 81.0 |  | 76.5 | 73.5 | 79.2 |  | 76.7 | 74.8 | 78.5 |
| 17 years old | 74.1 | 69.9 | 77.9 |  | 74.1 | 70.8 | 77.1 |  | 73.5 | 70.5 | 76.4 |
|  |  |  |  |  |  |  |  |  |  |  |  |
| **Race** |  |  |  |  |  |  |  |  |  |  |  |
| White | 80.0 | 75.2 | 84.0 |  | 82.1 | 79.8 | 84.1 |  | 81.7 | 79.3 | 83.9 |
| Black or African American | 73.4 | 70.5 | 76.0 |  | 66.4 | 61.6 | 70.8 |  | 68.0 | 64.6 | 71.3 |
| Hispanic/Latino | 76.8 | 73.9 | 79.4 |  | 73.9 | 70.4 | 77.1 |  | 73.2 | 70.1 | 76.1 |
| All Other Races | 75.6 | 72.1 | 78.9 |  | 75.6 | 72.6 | 78.4 |  | 77.5 | 74.2 | 80.4 |
|  |  |  |  |  |  |  |  |  |  |  |  |
| **Body weight** |  |  |  |  |  |  |  |  |  |  |  |
| Normal and underweight | 78.7 | 75.1 | 81.8 |  | 79.2 | 76.9 | 81.3 |  | 78.7 | 76.6 | 80.7 |
| Overweight | 78.3 | 74.9 | 81.4 |  | 74.6 | 71.5 | 77.4 |  | 76.5 | 74.3 | 78.6 |
| Obesity | 74.1 | 71.0 | 76.9 |  | 71.8 | 68.3 | 74.9 |  | 72.6 | 70.5 | 74.6 |
|  |  |  |  |  |  |  |  |  |  |  |  |
| **Recreational screen time per day** |  |  |  |  |  |  |  |  |  |  |  |
| No more than 2 hours | 79.3 | 73.8 | 83.9 |  | 77.6 | 73.8 | 80.9 |  | 78.0 | 75.6 | 80.3 |
| More than 2 hours | 77.2 | 75.0 | 79.3 |  | 77.1 | 75.1 | 79.0 |  | 77.2 | 75.2 | 79.0 |

**Supplementary Table 3. Weighted prevalence of at least two days of moderate to vigorous physical activity per week across different survey years.**

|  | **2005** | | |  | **2007** | | |  | **2009** | | |
| --- | --- | --- | --- | --- | --- | --- | --- | --- | --- | --- | --- |
|  | **%** | **95%CI** | |  | **%** | **95%CI** | |  | **%** | **95%CI** | |
| **Overall** | 64.7 | 62.8 | 66.5 |  | 65.7 | 64.0 | 67.5 |  | 67.5 | 65.4 | 69.5 |
|  |  |  |  |  |  |  |  |  |  |  |  |
| **Sex** |  |  |  |  |  |  |  |  |  |  |  |
| Female | 57.1 | 54.8 | 59.3 |  | 57.5 | 54.9 | 60.1 |  | 59.0 | 56.7 | 61.3 |
| Male | 72.3 | 70.1 | 74.5 |  | 73.9 | 72.1 | 75.6 |  | 75.4 | 72.5 | 78.0 |
|  |  |  |  |  |  |  |  |  |  |  |  |
| **Age** |  |  |  |  |  |  |  |  |  |  |  |
| 14 years old | 67.4 | 64.1 | 70.5 |  | 67.9 | 64.5 | 71.2 |  | 68.9 | 65.8 | 71.7 |
| 15 years old | 65.1 | 62.1 | 68.0 |  | 68.0 | 65.6 | 70.2 |  | 68.4 | 65.5 | 71.1 |
| 16 years old | 65.0 | 62.5 | 67.3 |  | 64.8 | 62.0 | 67.4 |  | 68.3 | 65.8 | 70.7 |
| 17 years old | 62.8 | 60.5 | 65.0 |  | 63.3 | 61.1 | 65.4 |  | 65.1 | 62.4 | 67.7 |
|  |  |  |  |  |  |  |  |  |  |  |  |
| **Race** |  |  |  |  |  |  |  |  |  |  |  |
| White | 66.9 | 64.8 | 68.9 |  | 68.7 | 66.4 | 70.8 |  | 71.0 | 68.4 | 73.4 |
| Black or African American | 58.0 | 54.6 | 61.3 |  | 59.2 | 56.5 | 61.8 |  | 60.6 | 57.0 | 64.1 |
| Hispanic/Latino | 62.5 | 58.9 | 66.0 |  | 61.7 | 58.4 | 64.9 |  | 64.2 | 61.9 | 66.4 |
| All Other Races | 64.5 | 59.6 | 69.1 |  | 64.8 | 59.6 | 69.7 |  | 62.8 | 57.8 | 67.4 |
|  |  |  |  |  |  |  |  |  |  |  |  |
| **Body weight** |  |  |  |  |  |  |  |  |  |  |  |
| Normal and underweight | 65.6 | 63.7 | 67.3 |  | 66.9 | 64.6 | 69.0 |  | 68.9 | 66.7 | 71.1 |
| Overweight | 63.4 | 60.0 | 66.8 |  | 63.9 | 60.9 | 66.9 |  | 66.2 | 63.1 | 69.1 |
| Obesity | 61.9 | 58.7 | 65.0 |  | 62.1 | 59.3 | 64.9 |  | 61.2 | 57.4 | 64.9 |
|  |  |  |  |  |  |  |  |  |  |  |  |
| **Recreational screen time per day** |  |  |  |  |  |  |  |  |  |  |  |
| No more than 2 hours | 68.5 | 65.9 | 70.9 |  | 69.2 | 65.9 | 72.4 |  | 72.2 | 69.1 | 75.2 |
| More than 2 hours | 62.9 | 60.9 | 64.9 |  | 64.1 | 62.3 | 65.8 |  | 65.1 | 63.2 | 67.0 |
|  | **2011** | | |  | **2013** | | |  | **2015** | | |
|  | **%** | **95%CI** | |  | **%** | **95%CI** | |  | **%** | **95%CI** | |
| **Overall** | 79.4 | 77.9 | 80.8 |  | 78.4 | 76.4 | 80.2 |  | 79.6 | 77.4 | 81.7 |
|  |  |  |  |  |  |  |  |  |  |  |  |
| **Sex** |  |  |  |  |  |  |  |  |  |  |  |
| Female | 73.6 | 71.6 | 75.5 |  | 71.9 | 69.2 | 74.6 |  | 74.8 | 71.8 | 77.7 |
| Male | 85.0 | 83.5 | 86.3 |  | 85.0 | 83.4 | 86.4 |  | 84.3 | 81.9 | 86.4 |
|  |  |  |  |  |  |  |  |  |  |  |  |
| **Age** |  |  |  |  |  |  |  |  |  |  |  |
| 14 years old | 80.3 | 77.2 | 83.0 |  | 83.6 | 79.8 | 86.7 |  | 83.0 | 80.0 | 85.6 |
| 15 years old | 81.7 | 79.6 | 83.7 |  | 81.4 | 79.1 | 83.5 |  | 82.2 | 79.4 | 84.6 |
| 16 years old | 78.4 | 76.5 | 80.1 |  | 78.0 | 75.8 | 80.0 |  | 77.8 | 75.1 | 80.3 |
| 17 years old | 77.7 | 75.7 | 79.6 |  | 73.7 | 70.7 | 76.4 |  | 77.4 | 74.3 | 80.2 |
|  |  |  |  |  |  |  |  |  |  |  |  |
| **Race** |  |  |  |  |  |  |  |  |  |  |  |
| White | 82.4 | 80.5 | 84.1 |  | 81.5 | 78.8 | 83.9 |  | 82.6 | 79.6 | 85.3 |
| Black or African American | 74.2 | 71.1 | 77.1 |  | 71.1 | 69.0 | 73.2 |  | 74.0 | 69.4 | 78.1 |
| Hispanic/Latino | 76.3 | 73.9 | 78.4 |  | 76.3 | 73.1 | 79.3 |  | 77.3 | 74.8 | 79.6 |
| All Other Races | 76.0 | 72.3 | 79.4 |  | 75.5 | 72.2 | 78.6 |  | 76.5 | 72.1 | 80.4 |
|  |  |  |  |  |  |  |  |  |  |  |  |
| **Body weight** |  |  |  |  |  |  |  |  |  |  |  |
| Normal and underweight | 80.1 | 78.5 | 81.7 |  | 79.2 | 77.5 | 80.9 |  | 80.7 | 78.3 | 82.9 |
| Overweight | 80.9 | 78.7 | 82.8 |  | 78.6 | 75.4 | 81.6 |  | 78.0 | 74.9 | 80.9 |
| Obesity | 73.7 | 70.0 | 77.1 |  | 73.7 | 69.4 | 77.6 |  | 76.3 | 73.3 | 79.1 |
|  |  |  |  |  |  |  |  |  |  |  |  |
| **Recreational screen time per day** |  |  |  |  |  |  |  |  |  |  |  |
| No more than 2 hours | 83.0 | 80.8 | 85.1 |  | 83.2 | 80.2 | 85.8 |  | 82.0 | 78.0 | 85.4 |
| More than 2 hours | 77.8 | 76.5 | 79.1 |  | 76.5 | 74.3 | 78.6 |  | 78.6 | 76.7 | 80.3 |
|  | **2017** | | |  | **2019** | | |  | **2021** | | |
|  | **%** | **95%CI** | |  | **%** | **95%CI** | |  | **%** | **95%CI** | |
| **Overall** | 77.9 | 74.8 | 80.7 |  | 77.3 | 75.1 | 79.4 |  | 77.4 | 75.5 | 79.1 |
|  |  |  |  |  |  |  |  |  |  |  |  |
| **Sex** |  |  |  |  |  |  |  |  |  |  |  |
| Female | 72.0 | 67.4 | 76.2 |  | 73.0 | 70.2 | 75.6 |  | 72.4 | 70.2 | 74.6 |
| Male | 84.0 | 82.5 | 85.5 |  | 81.5 | 79.2 | 83.5 |  | 81.9 | 80.1 | 83.6 |
|  |  |  |  |  |  |  |  |  |  |  |  |
| **Age** |  |  |  |  |  |  |  |  |  |  |  |
| 14 years old | 82.4 | 79.3 | 85.2 |  | 78.4 | 74.2 | 82.2 |  | 81.5 | 79.0 | 83.7 |
| 15 years old | 80.0 | 76.8 | 82.9 |  | 80.6 | 78.2 | 82.8 |  | 78.4 | 75.4 | 81.0 |
| 16 years old | 77.4 | 73.4 | 81.0 |  | 76.5 | 73.5 | 79.2 |  | 76.7 | 74.8 | 78.5 |
| 17 years old | 74.1 | 69.9 | 77.9 |  | 74.1 | 70.8 | 77.1 |  | 73.5 | 70.5 | 76.4 |
|  |  |  |  |  |  |  |  |  |  |  |  |
| **Race** |  |  |  |  |  |  |  |  |  |  |  |
| White | 80.0 | 75.2 | 84.0 |  | 82.1 | 79.8 | 84.1 |  | 81.7 | 79.3 | 83.9 |
| Black or African American | 73.4 | 70.5 | 76.0 |  | 66.4 | 61.6 | 70.8 |  | 68.0 | 64.6 | 71.3 |
| Hispanic/Latino | 76.8 | 73.9 | 79.4 |  | 73.9 | 70.4 | 77.1 |  | 73.2 | 70.1 | 76.1 |
| All Other Races | 75.6 | 72.1 | 78.9 |  | 75.6 | 72.6 | 78.4 |  | 77.5 | 74.2 | 80.4 |
|  |  |  |  |  |  |  |  |  |  |  |  |
| **Body weight** |  |  |  |  |  |  |  |  |  |  |  |
| Normal and underweight | 78.7 | 75.1 | 81.8 |  | 79.2 | 76.9 | 81.3 |  | 78.7 | 76.6 | 80.7 |
| Overweight | 78.3 | 74.9 | 81.4 |  | 74.6 | 71.5 | 77.4 |  | 76.5 | 74.3 | 78.6 |
| Obesity | 74.1 | 71.0 | 76.9 |  | 71.8 | 68.3 | 74.9 |  | 72.6 | 70.5 | 74.6 |
|  |  |  |  |  |  |  |  |  |  |  |  |
| **Recreational screen time per day** |  |  |  |  |  |  |  |  |  |  |  |
| No more than 2 hours | 79.3 | 73.8 | 83.9 |  | 77.6 | 73.8 | 80.9 |  | 78.0 | 75.6 | 80.3 |
| More than 2 hours | 77.2 | 75.0 | 79.3 |  | 77.1 | 75.1 | 79.0 |  | 77.2 | 75.2 | 79.0 |

**Supplementary Table 4. Weighted prevalence of at least three days of moderate to vigorous physical activity per week across different survey years.**

|  | **2005** | | |  | **2007** | | |  | **2009** | | |
| --- | --- | --- | --- | --- | --- | --- | --- | --- | --- | --- | --- |
|  | **%** | **95%CI** | |  | **%** | **95%CI** | |  | **%** | **95%CI** | |
| **Overall** | 54.2 | 52.3 | 56.1 |  | 55.3 | 53.2 | 57.4 |  | 56.7 | 54.4 | 59.0 |
|  |  |  |  |  |  |  |  |  |  |  |  |
| **Sex** |  |  |  |  |  |  |  |  |  |  |  |
| Female | 45.4 | 42.9 | 47.8 |  | 45.7 | 42.9 | 48.6 |  | 45.7 | 42.9 | 48.6 |
| Male | 63.0 | 60.7 | 65.3 |  | 45.7 | 42.9 | 48.6 |  | 45.7 | 42.9 | 48.6 |
|  |  |  |  |  |  |  |  |  |  |  |  |
| **Age** |  |  |  |  |  |  |  |  |  |  |  |
| 14 years old | 56.5 | 52.5 | 60.5 |  | 57.6 | 53.9 | 61.2 |  | 57.6 | 53.9 | 61.2 |
| 15 years old | 54.5 | 51.6 | 57.4 |  | 57.6 | 53.9 | 61.2 |  | 58.4 | 55.4 | 61.3 |
| 16 years old | 54.2 | 51.7 | 56.8 |  | 57.6 | 53.9 | 61.2 |  | 57.7 | 55.3 | 60.1 |
| 17 years old | 52.7 | 50.4 | 55.1 |  | 57.6 | 53.9 | 61.2 |  | 53.5 | 50.4 | 56.5 |
|  |  |  |  |  |  |  |  |  |  |  |  |
| **Race** |  |  |  |  |  |  |  |  |  |  |  |
| White | 56.8 | 54.6 | 59.0 |  | 58.1 | 55.3 | 60.9 |  | 60.3 | 57.2 | 63.4 |
| Black or African American | 47.7 | 44.1 | 51.3 |  | 50.1 | 47.6 | 52.5 |  | 50.8 | 47.6 | 53.9 |
| Hispanic/Latino | 50.8 | 47.8 | 53.8 |  | 50.9 | 48.1 | 53.7 |  | 52.6 | 50.0 | 55.1 |
| All Other Races | 52.3 | 46.8 | 57.7 |  | 54.1 | 48.8 | 59.2 |  | 51.3 | 46.4 | 56.3 |
|  |  |  |  |  |  |  |  |  |  |  |  |
| **Body weight** |  |  |  |  |  |  |  |  |  |  |  |
| Normal and underweight | 55.2 | 53.3 | 57.0 |  | 56.9 | 54.3 | 59.5 |  | 58.0 | 55.5 | 60.6 |
| Overweight | 53.1 | 49.8 | 56.4 |  | 53.4 | 50.2 | 56.6 |  | 56.1 | 52.6 | 59.6 |
| Obesity | 50.6 | 47.4 | 53.8 |  | 49.6 | 46.3 | 53.0 |  | 49.9 | 46.4 | 53.5 |
|  |  |  |  |  |  |  |  |  |  |  |  |
| **Recreational screen time per day** |  |  |  |  |  |  |  |  |  |  |  |
| No more than 2 hours | 58.4 | 55.5 | 61.2 |  | 60.5 | 56.4 | 64.4 |  | 61.8 | 58.4 | 65.1 |
| More than 2 hours | 52.2 | 50.1 | 54.3 |  | 52.9 | 51.1 | 54.7 |  | 54.2 | 51.9 | 56.4 |
|  | **2011** | | |  | **2013** | | |  | **2015** | | |
|  | **%** | **95%CI** | |  | **%** | **95%CI** | |  | **%** | **95%CI** | |
| **Overall** | 70.4 | 68.4 | 72.3 |  | 68.6 | 66.3 | 70.8 |  | 70.8 | 67.9 | 73.5 |
|  |  |  |  |  |  |  |  |  |  |  |  |
| **Sex** |  |  |  |  |  |  |  |  |  |  |  |
| Female | 62.5 | 60.1 | 64.9 |  | 62.5 | 60.1 | 64.9 |  | 62.5 | 60.1 | 64.9 |
| Male | 62.5 | 60.1 | 64.9 |  | 62.5 | 60.1 | 64.9 |  | 62.5 | 60.1 | 64.9 |
|  |  |  |  |  |  |  |  |  |  |  |  |
| **Age** |  |  |  |  |  |  |  |  |  |  |  |
| 14 years old | 72.0 | 68.9 | 75.0 |  | 72.9 | 68.2 | 77.2 |  | 74.3 | 70.7 | 77.6 |
| 15 years old | 73.4 | 70.4 | 76.2 |  | 72.3 | 69.4 | 74.9 |  | 74.7 | 71.1 | 77.9 |
| 16 years old | 69.0 | 66.5 | 71.5 |  | 68.1 | 65.7 | 70.5 |  | 68.3 | 64.9 | 71.6 |
| 17 years old | 68.0 | 65.6 | 70.4 |  | 63.7 | 60.6 | 66.7 |  | 67.5 | 64.4 | 70.5 |
|  |  |  |  |  |  |  |  |  |  |  |  |
| **Race** |  |  |  |  |  |  |  |  |  |  |  |
| White | 74.2 | 71.7 | 76.5 |  | 71.6 | 68.5 | 74.6 |  | 74.8 | 71.0 | 78.3 |
| Black or African American | 63.7 | 59.9 | 67.4 |  | 60.8 | 57.8 | 63.7 |  | 64.1 | 59.0 | 69.0 |
| Hispanic/Latino | 66.4 | 63.6 | 69.1 |  | 67.0 | 63.2 | 70.5 |  | 67.3 | 64.5 | 70.0 |
| All Other Races | 66.4 | 62.6 | 70.0 |  | 66.4 | 62.4 | 70.2 |  | 65.8 | 60.4 | 70.7 |
|  |  |  |  |  |  |  |  |  |  |  |  |
| **Body weight** |  |  |  |  |  |  |  |  |  |  |  |
| Normal and underweight | 71.8 | 69.7 | 73.9 |  | 69.9 | 67.7 | 72.0 |  | 72.1 | 68.8 | 75.1 |
| Overweight | 69.9 | 67.0 | 72.6 |  | 69.2 | 65.9 | 72.4 |  | 68.1 | 64.5 | 71.4 |
| Obesity | 63.3 | 59.3 | 67.2 |  | 61.3 | 56.3 | 66.1 |  | 67.3 | 63.7 | 70.7 |
|  |  |  |  |  |  |  |  |  |  |  |  |
| **Recreational screen time per day** |  |  |  |  |  |  |  |  |  |  |  |
| No more than 2 hours | 74.6 | 71.6 | 77.4 |  | 74.3 | 71.1 | 77.3 |  | 74.6 | 70.1 | 78.7 |
| More than 2 hours | 68.6 | 66.8 | 70.3 |  | 66.4 | 63.9 | 68.9 |  | 69.0 | 66.7 | 71.2 |
|  | **2017** | | |  | **2019** | | |  | **2021** | | |
|  | **%** | **95%CI** | |  | **%** | **95%CI** | |  | **%** | **95%CI** | |
| **Overall** | 68.5 | 64.9 | 72.0 |  | 67.4 | 65.0 | 69.7 |  | 67.6 | 65.4 | 69.7 |
|  |  |  |  |  |  |  |  |  |  |  |  |
| **Sex** |  |  |  |  |  |  |  |  |  |  |  |
| Female | 60.4 | 55.3 | 65.2 |  | 60.4 | 55.3 | 65.2 |  | 60.3 | 57.7 | 62.8 |
| Male | 60.4 | 55.3 | 65.2 |  | 60.4 | 55.3 | 65.2 |  | 74.4 | 72.1 | 76.5 |
|  |  |  |  |  |  |  |  |  |  |  |  |
| **Age** |  |  |  |  |  |  |  |  |  |  |  |
| 14 years old | 74.3 | 69.5 | 78.5 |  | 69.8 | 65.4 | 73.8 |  | 70.9 | 68.1 | 73.5 |
| 15 years old | 71.3 | 67.7 | 74.7 |  | 71.0 | 68.3 | 73.5 |  | 70.0 | 67.0 | 73.0 |
| 16 years old | 67.1 | 62.4 | 71.6 |  | 65.8 | 62.5 | 68.9 |  | 67.3 | 64.7 | 69.8 |
| 17 years old | 64.4 | 60.2 | 68.4 |  | 64.3 | 61.4 | 67.2 |  | 62.6 | 58.8 | 66.2 |
|  |  |  |  |  |  |  |  |  |  |  |  |
| **Race** |  |  |  |  |  |  |  |  |  |  |  |
| White | 70.4 | 65.0 | 75.3 |  | 72.6 | 70.2 | 74.9 |  | 73.4 | 70.2 | 76.3 |
| Black or African American | 63.3 | 59.3 | 67.1 |  | 55.8 | 51.3 | 60.2 |  | 55.7 | 52.1 | 59.3 |
| Hispanic/Latino | 68.1 | 64.9 | 71.2 |  | 63.3 | 59.7 | 66.8 |  | 62.5 | 59.7 | 65.2 |
| All Other Races | 66.8 | 62.9 | 70.5 |  | 66.3 | 63.4 | 69.1 |  | 66.4 | 62.9 | 69.7 |
|  |  |  |  |  |  |  |  |  |  |  |  |
| **Body weight** |  |  |  |  |  |  |  |  |  |  |  |
| Normal and underweight | 69.8 | 65.5 | 73.8 |  | 70.0 | 67.5 | 72.5 |  | 69.8 | 67.3 | 72.1 |
| Overweight | 67.8 | 64.2 | 71.2 |  | 63.8 | 61.0 | 66.6 |  | 64.8 | 61.9 | 67.5 |
| Obesity | 63.5 | 59.8 | 67.0 |  | 59.9 | 56.3 | 63.3 |  | 61.3 | 58.3 | 64.2 |
|  |  |  |  |  |  |  |  |  |  |  |  |
| **Recreational screen time per day** |  |  |  |  |  |  |  |  |  |  |  |
| No more than 2 hours | 70.0 | 63.3 | 75.9 |  | 70.3 | 66.5 | 73.9 |  | 71.0 | 67.7 | 74.2 |
| More than 2 hours | 67.8 | 65.3 | 70.3 |  | 66.1 | 63.8 | 68.2 |  | 66.5 | 64.3 | 68.7 |

**Supplementary Table 5. Weighted prevalence of at least four days of moderate to vigorous physical activity per week across different survey years.**

|  | **2005** | | |  | **2007** | | |  | **2009** | | |
| --- | --- | --- | --- | --- | --- | --- | --- | --- | --- | --- | --- |
|  | **%** | **95%CI** | |  | **%** | **95%CI** | |  | **%** | **95%CI** | |
| **Overall** | 44.1 | 42.1 | 46.0 |  | 43.9 | 41.7 | 46.2 |  | 46.5 | 44.5 | 48.5 |
|  |  |  |  |  |  |  |  |  |  |  |  |
| **Sex** |  |  |  |  |  |  |  |  |  |  |  |
| Female | 35.9 | 33.4 | 38.4 |  | 34.4 | 31.6 | 37.3 |  | 36.0 | 33.6 | 38.4 |
| Male | 52.3 | 50.0 | 54.5 |  | 53.4 | 50.9 | 55.8 |  | 56.2 | 53.6 | 58.9 |
|  |  |  |  |  |  |  |  |  |  |  |  |
| **Age** |  |  |  |  |  |  |  |  |  |  |  |
| 14 years old | 45.5 | 41.4 | 49.6 |  | 46.1 | 42.5 | 49.8 |  | 47.0 | 44.0 | 50.0 |
| 15 years old | 45.0 | 42.2 | 47.9 |  | 45.2 | 42.1 | 48.4 |  | 49.3 | 46.2 | 52.3 |
| 16 years old | 44.2 | 42.0 | 46.5 |  | 44.7 | 41.6 | 47.8 |  | 47.1 | 45.0 | 49.3 |
| 17 years old | 42.2 | 39.5 | 45.0 |  | 40.6 | 38.2 | 43.2 |  | 42.8 | 40.1 | 45.5 |
|  |  |  |  |  |  |  |  |  |  |  |  |
| **Race** |  |  |  |  |  |  |  |  |  |  |  |
| White | 46.9 | 44.5 | 49.4 |  | 46.2 | 43.2 | 49.3 |  | 49.8 | 47.2 | 52.4 |
| Black or African American | 37.0 | 33.8 | 40.3 |  | 40.2 | 37.7 | 42.7 |  | 41.8 | 38.9 | 44.7 |
| Hispanic/Latino | 41.8 | 38.6 | 45.2 |  | 39.6 | 36.5 | 42.6 |  | 42.0 | 39.3 | 44.8 |
| All Other Races | 39.5 | 34.7 | 44.6 |  | 43.3 | 39.0 | 47.8 |  | 41.9 | 37.0 | 47.0 |
|  |  |  |  |  |  |  |  |  |  |  |  |
| **Body weight** |  |  |  |  |  |  |  |  |  |  |  |
| Normal and underweight | 45.6 | 43.5 | 47.7 |  | 45.7 | 42.9 | 48.4 |  | 47.8 | 45.5 | 50.1 |
| Overweight | 41.9 | 39.0 | 44.9 |  | 41.3 | 38.2 | 44.4 |  | 46.6 | 43.4 | 49.8 |
| Obesity | 38.9 | 36.0 | 41.9 |  | 38.2 | 35.2 | 41.2 |  | 39.0 | 36.0 | 42.1 |
|  |  |  |  |  |  |  |  |  |  |  |  |
| **Recreational screen time per day** |  |  |  |  |  |  |  |  |  |  |  |
| No more than 2 hours | 50.3 | 47.0 | 53.5 |  | 49.5 | 45.4 | 53.7 |  | 52.5 | 49.3 | 55.8 |
| More than 2 hours | 41.2 | 39.1 | 43.3 |  | 41.3 | 39.3 | 43.2 |  | 43.5 | 41.4 | 45.6 |
|  | **2011** | | |  | **2013** | | |  | **2015** | | |
|  | **%** | **95%CI** | |  | **%** | **95%CI** | |  | **%** | **95%CI** | |
| **Overall** | 60.1 | 57.9 | 62.1 |  | 57.2 | 54.9 | 59.5 |  | 59.4 | 56.5 | 62.2 |
|  |  |  |  |  |  |  |  |  |  |  |  |
| **Sex** |  |  |  |  |  |  |  |  |  |  |  |
| Female | 50.6 | 48.0 | 53.2 |  | 47.3 | 44.0 | 50.6 |  | 51.0 | 46.8 | 55.2 |
| Male | 69.1 | 66.9 | 71.3 |  | 67.4 | 65.9 | 69.0 |  | 67.4 | 64.6 | 70.1 |
|  |  |  |  |  |  |  |  |  |  |  |  |
| **Age** |  |  |  |  |  |  |  |  |  |  |  |
| 14 years old | 61.6 | 58.2 | 64.9 |  | 62.5 | 57.5 | 67.2 |  | 62.9 | 59.4 | 66.3 |
| 15 years old | 63.9 | 60.7 | 67.0 |  | 59.3 | 56.2 | 62.3 |  | 63.9 | 59.8 | 67.8 |
| 16 years old | 58.6 | 55.7 | 61.5 |  | 56.3 | 53.8 | 58.7 |  | 57.0 | 53.7 | 60.2 |
| 17 years old | 56.8 | 54.6 | 59.1 |  | 54.0 | 50.9 | 57.2 |  | 55.4 | 52.3 | 58.4 |
|  |  |  |  |  |  |  |  |  |  |  |  |
| **Race** |  |  |  |  |  |  |  |  |  |  |  |
| White | 64.0 | 61.4 | 66.6 |  | 60.3 | 57.3 | 63.3 |  | 63.4 | 59.5 | 67.2 |
| Black or African American | 52.9 | 48.7 | 57.1 |  | 49.9 | 46.6 | 53.1 |  | 54.0 | 48.6 | 59.2 |
| Hispanic/Latino | 55.9 | 53.1 | 58.6 |  | 54.9 | 51.6 | 58.2 |  | 55.0 | 52.1 | 57.8 |
| All Other Races | 56.0 | 51.8 | 60.1 |  | 55.2 | 51.2 | 59.1 |  | 54.5 | 49.5 | 59.4 |
|  |  |  |  |  |  |  |  |  |  |  |  |
| **Body weight** |  |  |  |  |  |  |  |  |  |  |  |
| Normal and underweight | 61.3 | 59.1 | 63.5 |  | 59.1 | 56.9 | 61.2 |  | 60.6 | 57.4 | 63.8 |
| Overweight | 60.0 | 56.9 | 62.9 |  | 56.1 | 52.4 | 59.7 |  | 57.2 | 53.2 | 61.2 |
| Obesity | 53.2 | 49.4 | 56.9 |  | 49.2 | 44.2 | 54.1 |  | 55.5 | 51.9 | 59.2 |
|  |  |  |  |  |  |  |  |  |  |  |  |
| **Recreational screen time per day** |  |  |  |  |  |  |  |  |  |  |  |
| No more than 2 hours | 65.9 | 62.7 | 69.0 |  | 64.1 | 60.7 | 67.4 |  | 63.5 | 59.5 | 67.2 |
| More than 2 hours | 57.5 | 55.6 | 59.3 |  | 54.6 | 52.1 | 57.0 |  | 57.5 | 54.9 | 60.0 |
|  | **2017** | | |  | **2019** | | |  | **2021** | | |
|  | **%** | **95%CI** | |  | **%** | **95%CI** | |  | **%** | **95%CI** | |
| **Overall** | 56.9 | 53.6 | 60.1 |  | 55.5 | 53.0 | 57.9 |  | 55.8 | 53.6 | 58.0 |
|  |  |  |  |  |  |  |  |  |  |  |  |
| **Sex** |  |  |  |  |  |  |  |  |  |  |  |
| Female | 47.1 | 43.0 | 51.3 |  | 47.7 | 44.8 | 50.6 |  | 47.2 | 44.8 | 49.6 |
| Male | 67.0 | 64.8 | 69.2 |  | 63.0 | 60.3 | 65.7 |  | 63.8 | 61.1 | 66.3 |
|  |  |  |  |  |  |  |  |  |  |  |  |
| **Age** |  |  |  |  |  |  |  |  |  |  |  |
| 14 years old | 61.9 | 56.7 | 66.9 |  | 59.2 | 54.3 | 63.9 |  | 59.8 | 56.5 | 63.0 |
| 15 years old | 59.9 | 56.6 | 63.0 |  | 59.8 | 56.5 | 62.9 |  | 58.7 | 55.6 | 61.7 |
| 16 years old | 54.7 | 50.4 | 58.9 |  | 52.9 | 49.8 | 56.0 |  | 54.9 | 52.6 | 57.3 |
| 17 years old | 53.8 | 50.0 | 57.4 |  | 51.9 | 48.9 | 54.8 |  | 50.4 | 47.1 | 53.7 |
|  |  |  |  |  |  |  |  |  |  |  |  |
| **Race** |  |  |  |  |  |  |  |  |  |  |  |
| White | 59.0 | 54.3 | 63.6 |  | 60.8 | 58.2 | 63.3 |  | 61.9 | 58.3 | 65.3 |
| Black or African American | 52.3 | 47.6 | 57.0 |  | 44.9 | 40.0 | 49.9 |  | 44.7 | 40.4 | 49.0 |
| Hispanic/Latino | 55.3 | 52.2 | 58.4 |  | 52.0 | 48.4 | 55.6 |  | 50.0 | 47.8 | 52.2 |
| All Other Races | 55.2 | 51.2 | 59.2 |  | 50.8 | 47.3 | 54.3 |  | 54.1 | 50.6 | 57.5 |
|  |  |  |  |  |  |  |  |  |  |  |  |
| **Body weight** |  |  |  |  |  |  |  |  |  |  |  |
| Normal and underweight | 58.5 | 54.6 | 62.3 |  | 58.5 | 55.7 | 61.2 |  | 58.2 | 55.4 | 60.9 |
| Overweight | 55.4 | 51.9 | 58.8 |  | 51.2 | 48.1 | 54.3 |  | 53.3 | 50.4 | 56.2 |
| Obesity | 50.9 | 47.3 | 54.6 |  | 46.8 | 43.2 | 50.5 |  | 48.3 | 45.3 | 51.2 |
|  |  |  |  |  |  |  |  |  |  |  |  |
| **Recreational screen time per day** |  |  |  |  |  |  |  |  |  |  |  |
| No more than 2 hours | 59.5 | 53.5 | 65.3 |  | 60.6 | 56.9 | 64.3 |  | 60.8 | 57.2 | 64.3 |
| More than 2 hours | 55.6 | 53.1 | 58.0 |  | 53.0 | 50.6 | 55.4 |  | 54.3 | 52.0 | 56.5 |

**Supplementary Table 6. Weighted prevalence of at least five days of moderate to vigorous physical activity per week across different survey years.**

|  | **2005** | | |  | **2007** | | |  | **2009** | | |
| --- | --- | --- | --- | --- | --- | --- | --- | --- | --- | --- | --- |
|  | **%** | **95%CI** | |  | **%** | **95%CI** | |  | **%** | **95%CI** | |
| **Overall** | 36.6 | 34.6 | 38.5 |  | 35.8 | 33.7 | 37.9 |  | 37.9 | 36.0 | 39.7 |
|  |  |  |  |  |  |  |  |  |  |  |  |
| **Sex** |  |  |  |  |  |  |  |  |  |  |  |
| Female | 28.7 | 26.4 | 31.1 |  | 26.6 | 24.1 | 29.3 |  | 28.5 | 26.3 | 30.8 |
| Male | 44.5 | 42.2 | 46.8 |  | 44.9 | 42.4 | 47.4 |  | 46.5 | 44.6 | 48.5 |
|  |  |  |  |  |  |  |  |  |  |  |  |
| **Age** |  |  |  |  |  |  |  |  |  |  |  |
| 14 years old | 36.7 | 32.8 | 40.9 |  | 37.2 | 33.9 | 40.6 |  | 39.0 | 36.3 | 41.8 |
| 15 years old | 38.2 | 35.4 | 41.2 |  | 37.3 | 34.5 | 40.3 |  | 39.9 | 37.5 | 42.4 |
| 16 years old | 36.5 | 34.4 | 38.6 |  | 36.1 | 32.9 | 39.4 |  | 38.8 | 36.5 | 41.2 |
| 17 years old | 34.6 | 32.3 | 37.1 |  | 33.1 | 30.6 | 35.6 |  | 34.2 | 31.8 | 36.6 |
|  |  |  |  |  |  |  |  |  |  |  |  |
| **Race** |  |  |  |  |  |  |  |  |  |  |  |
| White | 39.4 | 37.0 | 41.9 |  | 38.3 | 35.4 | 41.3 |  | 41.2 | 39.0 | 43.4 |
| Black or African American | 30.0 | 27.1 | 33.2 |  | 31.9 | 29.9 | 33.9 |  | 33.8 | 30.7 | 37.0 |
| Hispanic/Latino | 33.7 | 30.6 | 37.0 |  | 31.6 | 28.8 | 34.5 |  | 33.0 | 30.7 | 35.4 |
| All Other Races | 31.9 | 27.0 | 37.2 |  | 33.3 | 29.5 | 37.4 |  | 32.7 | 27.6 | 38.3 |
|  |  |  |  |  |  |  |  |  |  |  |  |
| **Body weight** |  |  |  |  |  |  |  |  |  |  |  |
| Normal and underweight | 38.2 | 36.1 | 40.4 |  | 37.3 | 34.9 | 39.8 |  | 39.2 | 37.0 | 41.3 |
| Overweight | 33.7 | 30.7 | 36.9 |  | 34.2 | 31.2 | 37.3 |  | 38.0 | 35.4 | 40.7 |
| Obesity | 31.6 | 29.0 | 34.4 |  | 30.0 | 27.1 | 33.0 |  | 30.3 | 27.7 | 33.1 |
|  |  |  |  |  |  |  |  |  |  |  |  |
| **Recreational screen time per day** |  |  |  |  |  |  |  |  |  |  |  |
| No more than 2 hours | 42.4 | 39.0 | 45.9 |  | 41.3 | 37.4 | 45.3 |  | 44.4 | 41.7 | 47.1 |
| More than 2 hours | 33.8 | 31.9 | 35.8 |  | 33.2 | 31.3 | 35.1 |  | 34.6 | 32.6 | 36.7 |
|  | **2011** | | |  | **2013** | | |  | **2015** | | |
|  | **%** | **95%CI** | |  | **%** | **95%CI** | |  | **%** | **95%CI** | |
| **Overall** | 50.0 | 47.9 | 52.0 |  | 47.4 | 45.3 | 49.5 |  | 49.3 | 46.6 | 52.1 |
|  |  |  |  |  |  |  |  |  |  |  |  |
| **Sex** |  |  |  |  |  |  |  |  |  |  |  |
| Female | 39.7 | 37.1 | 42.4 |  | 37.5 | 34.8 | 40.2 |  | 40.1 | 36.5 | 43.8 |
| Male | 59.8 | 57.7 | 61.8 |  | 57.6 | 55.6 | 59.6 |  | 58.3 | 55.3 | 61.1 |
|  |  |  |  |  |  |  |  |  |  |  |  |
| **Age** |  |  |  |  |  |  |  |  |  |  |  |
| 14 years old | 53.0 | 49.1 | 56.7 |  | 53.6 | 48.8 | 58.4 |  | 50.1 | 46.7 | 53.5 |
| 15 years old | 53.0 | 49.7 | 56.2 |  | 49.2 | 46.0 | 52.5 |  | 54.2 | 50.5 | 57.9 |
| 16 years old | 48.9 | 46.4 | 51.5 |  | 46.3 | 44.1 | 48.5 |  | 47.2 | 44.0 | 50.4 |
| 17 years old | 46.4 | 44.4 | 48.5 |  | 44.2 | 41.5 | 47.0 |  | 45.9 | 42.7 | 49.2 |
|  |  |  |  |  |  |  |  |  |  |  |  |
| **Race** |  |  |  |  |  |  |  |  |  |  |  |
| White | 52.9 | 50.2 | 55.7 |  | 50.1 | 47.3 | 52.9 |  | 53.2 | 49.6 | 56.7 |
| Black or African American | 45.5 | 41.5 | 49.5 |  | 41.7 | 38.7 | 44.7 |  | 44.5 | 39.7 | 49.4 |
| Hispanic/Latino | 46.0 | 43.2 | 48.9 |  | 45.3 | 41.9 | 48.7 |  | 44.4 | 41.5 | 47.4 |
| All Other Races | 47.2 | 43.4 | 51.0 |  | 45.3 | 40.9 | 49.7 |  | 46.3 | 41.8 | 50.9 |
|  |  |  |  |  |  |  |  |  |  |  |  |
| **Body weight** |  |  |  |  |  |  |  |  |  |  |  |
| Normal and underweight | 51.4 | 49.0 | 53.9 |  | 49.2 | 47.2 | 51.2 |  | 50.7 | 47.8 | 53.6 |
| Overweight | 49.0 | 45.9 | 52.2 |  | 47.2 | 43.8 | 50.6 |  | 47.5 | 43.5 | 51.6 |
| Obesity | 42.9 | 39.6 | 46.3 |  | 38.5 | 34.7 | 42.4 |  | 44.5 | 40.8 | 48.4 |
|  |  |  |  |  |  |  |  |  |  |  |  |
| **Recreational screen time per day** |  |  |  |  |  |  |  |  |  |  |  |
| No more than 2 hours | 57.3 | 54.0 | 60.4 |  | 55.1 | 52.1 | 58.0 |  | 53.6 | 50.1 | 57.1 |
| More than 2 hours | 46.7 | 45.0 | 48.5 |  | 44.5 | 42.3 | 46.7 |  | 47.4 | 44.8 | 50.0 |
|  | **2017** | | |  | **2019** | | |  | **2021** | | |
|  | **%** | **95%CI** | |  | **%** | **95%CI** | |  | **%** | **95%CI** | |
| **Overall** | 47.2 | 44.1 | 50.4 |  | 44.7 | 42.5 | 47.0 |  | 45.3 | 43.2 | 47.5 |
|  |  |  |  |  |  |  |  |  |  |  |  |
| **Sex** |  |  |  |  |  |  |  |  |  |  |  |
| Female | 37.2 | 33.6 | 41.0 |  | 36.2 | 33.7 | 38.7 |  | 35.8 | 33.4 | 38.3 |
| Male | 57.5 | 55.0 | 60.0 |  | 53.1 | 50.5 | 55.6 |  | 54.1 | 51.7 | 56.6 |
|  |  |  |  |  |  |  |  |  |  |  |  |
| **Age** |  |  |  |  |  |  |  |  |  |  |  |
| 14 years old | 50.6 | 45.7 | 55.5 |  | 47.3 | 43.4 | 51.3 |  | 47.9 | 44.1 | 51.7 |
| 15 years old | 50.3 | 47.1 | 53.5 |  | 49.3 | 45.9 | 52.8 |  | 48.6 | 45.6 | 51.5 |
| 16 years old | 45.8 | 41.8 | 49.8 |  | 42.7 | 39.9 | 45.5 |  | 43.3 | 40.9 | 45.7 |
| 17 years old | 43.9 | 40.4 | 47.5 |  | 40.9 | 37.9 | 43.9 |  | 41.9 | 38.8 | 45.0 |
|  |  |  |  |  |  |  |  |  |  |  |  |
| **Race** |  |  |  |  |  |  |  |  |  |  |  |
| White | 49.2 | 45.0 | 53.4 |  | 49.6 | 47.1 | 52.1 |  | 51.5 | 47.9 | 55.1 |
| Black or African American | 43.0 | 38.0 | 48.2 |  | 37.1 | 32.6 | 41.8 |  | 35.2 | 31.4 | 39.3 |
| Hispanic/Latino | 45.7 | 42.5 | 48.9 |  | 41.0 | 37.4 | 44.6 |  | 39.4 | 36.6 | 42.4 |
| All Other Races | 45.9 | 41.9 | 49.9 |  | 39.7 | 36.6 | 42.8 |  | 42.3 | 39.0 | 45.6 |
|  |  |  |  |  |  |  |  |  |  |  |  |
| **Body weight** |  |  |  |  |  |  |  |  |  |  |  |
| Normal and underweight | 49.0 | 45.3 | 52.7 |  | 48.2 | 45.7 | 50.7 |  | 48.0 | 45.6 | 50.4 |
| Overweight | 45.8 | 42.4 | 49.3 |  | 39.2 | 36.1 | 42.3 |  | 43.2 | 39.7 | 46.7 |
| Obesity | 40.3 | 36.7 | 44.1 |  | 35.3 | 31.9 | 38.8 |  | 36.3 | 33.8 | 38.9 |
|  |  |  |  |  |  |  |  |  |  |  |  |
| **Recreational screen time per day** |  |  |  |  |  |  |  |  |  |  |  |
| No more than 2 hours | 51.4 | 45.9 | 56.9 |  | 50.6 | 47.4 | 53.9 |  | 51.7 | 48.1 | 55.2 |
| More than 2 hours | 45.1 | 42.7 | 47.6 |  | 41.9 | 39.8 | 44.2 |  | 43.3 | 41.4 | 45.3 |

**Supplementary Table 7. Weighted prevalence of at least six days of moderate to vigorous physical activity per week across different survey years.**

|  | **2005** | | |  | **2007** | | |  | **2009** | | |
| --- | --- | --- | --- | --- | --- | --- | --- | --- | --- | --- | --- |
|  | **%** | **95%CI** | |  | **%** | **95%CI** | |  | **%** | **95%CI** | |
| **Overall** | 24.8 | 23.3 | 26.3 |  | 23.7 | 22.3 | 25.3 |  | 26.0 | 24.4 | 27.6 |
|  |  |  |  |  |  |  |  |  |  |  |  |
| **Sex** |  |  |  |  |  |  |  |  |  |  |  |
| Female | 17.9 | 16.6 | 19.4 |  | 16.0 | 13.9 | 18.3 |  | 17.8 | 16.1 | 19.6 |
| Male | 31.7 | 29.6 | 33.8 |  | 31.4 | 29.6 | 33.3 |  | 33.6 | 31.8 | 35.5 |
|  |  |  |  |  |  |  |  |  |  |  |  |
| **Age** |  |  |  |  |  |  |  |  |  |  |  |
| 14 years old | 23.4 | 20.1 | 27.0 |  | 25.6 | 22.8 | 28.7 |  | 27.1 | 24.4 | 30.0 |
| 15 years old | 27.2 | 25.0 | 29.6 |  | 23.8 | 21.8 | 25.9 |  | 26.7 | 24.6 | 29.0 |
| 16 years old | 24.1 | 22.1 | 26.1 |  | 24.6 | 22.4 | 26.9 |  | 27.1 | 24.9 | 29.4 |
| 17 years old | 23.4 | 21.4 | 25.6 |  | 21.8 | 19.9 | 23.9 |  | 23.6 | 21.4 | 25.9 |
|  |  |  |  |  |  |  |  |  |  |  |  |
| **Race** |  |  |  |  |  |  |  |  |  |  |  |
| White | 26.9 | 25.0 | 28.9 |  | 26.1 | 23.6 | 28.7 |  | 28.9 | 27.0 | 30.9 |
| Black or African American | 20.6 | 18.6 | 22.8 |  | 20.5 | 18.8 | 22.4 |  | 22.8 | 20.1 | 25.6 |
| Hispanic/Latino | 22.0 | 19.5 | 24.6 |  | 19.9 | 17.9 | 22.0 |  | 21.0 | 19.4 | 22.8 |
| All Other Races | 21.6 | 17.4 | 26.6 |  | 20.6 | 17.4 | 24.2 |  | 22.8 | 19.0 | 27.1 |
|  |  |  |  |  |  |  |  |  |  |  |  |
| **Body weight** |  |  |  |  |  |  |  |  |  |  |  |
| Normal and underweight | 26.3 | 24.5 | 28.1 |  | 24.7 | 22.9 | 26.5 |  | 27.5 | 25.7 | 29.4 |
| Overweight | 22.5 | 20.4 | 24.7 |  | 22.8 | 21.0 | 24.7 |  | 25.6 | 23.3 | 28.1 |
| Obesity | 20.2 | 18.1 | 22.5 |  | 20.0 | 17.9 | 22.3 |  | 18.1 | 16.0 | 20.3 |
|  |  |  |  |  |  |  |  |  |  |  |  |
| **Recreational screen time per day** |  |  |  |  |  |  |  |  |  |  |  |
| No more than 2 hours | 29.5 | 26.9 | 32.2 |  | 27.7 | 25.1 | 30.4 |  | 31.8 | 29.3 | 34.3 |
| More than 2 hours | 22.6 | 21.2 | 24.1 |  | 21.9 | 20.5 | 23.2 |  | 23.1 | 21.3 | 25.1 |
|  | **2011** | | |  | **2013** | | |  | **2015** | | |
|  | **%** | **95%CI** | |  | **%** | **95%CI** | |  | **%** | **95%CI** | |
| **Overall** | 36.9 | 34.7 | 39.2 |  | 34.1 | 32.6 | 35.7 |  | 34.3 | 32.1 | 36.5 |
|  |  |  |  |  |  |  |  |  |  |  |  |
| **Sex** |  |  |  |  |  |  |  |  |  |  |  |
| Female | 26.5 | 24.0 | 29.1 |  | 24.1 | 22.3 | 26.0 |  | 25.3 | 22.6 | 28.1 |
| Male | 46.9 | 44.5 | 49.2 |  | 44.5 | 42.5 | 46.4 |  | 43.1 | 40.6 | 45.6 |
|  |  |  |  |  |  |  |  |  |  |  |  |
| **Age** |  |  |  |  |  |  |  |  |  |  |  |
| 14 years old | 40.3 | 36.6 | 44.1 |  | 37.2 | 33.3 | 41.4 |  | 32.7 | 30.0 | 35.5 |
| 15 years old | 38.3 | 34.9 | 41.9 |  | 35.2 | 32.8 | 37.6 |  | 37.5 | 34.2 | 40.8 |
| 16 years old | 36.2 | 33.9 | 38.6 |  | 34.3 | 32.5 | 36.1 |  | 33.7 | 31.0 | 36.5 |
| 17 years old | 34.4 | 32.2 | 36.8 |  | 31.7 | 29.3 | 34.2 |  | 32.1 | 29.5 | 34.9 |
|  |  |  |  |  |  |  |  |  |  |  |  |
| **Race** |  |  |  |  |  |  |  |  |  |  |  |
| White | 39.6 | 36.7 | 42.7 |  | 36.6 | 34.5 | 38.7 |  | 37.5 | 34.7 | 40.4 |
| Black or African American | 33.1 | 30.3 | 35.9 |  | 30.9 | 28.3 | 33.7 |  | 29.7 | 25.8 | 33.8 |
| Hispanic/Latino | 33.3 | 31.3 | 35.4 |  | 30.9 | 28.5 | 33.4 |  | 29.9 | 27.1 | 32.9 |
| All Other Races | 33.9 | 30.7 | 37.2 |  | 31.9 | 28.5 | 35.5 |  | 33.2 | 28.8 | 37.9 |
|  |  |  |  |  |  |  |  |  |  |  |  |
| **Body weight** |  |  |  |  |  |  |  |  |  |  |  |
| Normal and underweight | 38.8 | 36.2 | 41.6 |  | 36.1 | 34.6 | 37.7 |  | 35.9 | 33.5 | 38.4 |
| Overweight | 34.2 | 31.0 | 37.5 |  | 32.4 | 29.2 | 35.7 |  | 31.8 | 28.3 | 35.4 |
| Obesity | 29.4 | 26.8 | 32.2 |  | 26.1 | 22.9 | 29.6 |  | 29.2 | 26.4 | 32.2 |
|  |  |  |  |  |  |  |  |  |  |  |  |
| **Recreational screen time per day** |  |  |  |  |  |  |  |  |  |  |  |
| No more than 2 hours | 44.6 | 41.2 | 48.1 |  | 42.8 | 39.9 | 45.7 |  | 39.7 | 36.8 | 42.8 |
| More than 2 hours | 33.5 | 31.7 | 35.3 |  | 30.8 | 29.3 | 32.3 |  | 31.8 | 29.7 | 34.0 |
|  | **2017** | | |  | **2019** | | |  | **2021** | | |
|  | **%** | **95%CI** | |  | **%** | **95%CI** | |  | **%** | **95%CI** | |
| **Overall** | 33.3 | 31.0 | 35.7 |  | 31.1 | 29.3 | 32.9 |  | 31.6 | 29.6 | 33.6 |
|  |  |  |  |  |  |  |  |  |  |  |  |
| **Sex** |  |  |  |  |  |  |  |  |  |  |  |
| Female | 23.1 | 21.0 | 25.5 |  | 22.7 | 20.8 | 24.7 |  | 21.9 | 20.1 | 23.9 |
| Male | 43.9 | 41.5 | 46.2 |  | 39.2 | 36.8 | 41.6 |  | 40.5 | 37.8 | 43.2 |
|  |  |  |  |  |  |  |  |  |  |  |  |
| **Age** |  |  |  |  |  |  |  |  |  |  |  |
| 14 years old | 33.8 | 29.8 | 38.0 |  | 33.7 | 30.2 | 37.4 |  | 32.5 | 29.1 | 36.0 |
| 15 years old | 36.6 | 34.0 | 39.3 |  | 33.5 | 31.2 | 35.9 |  | 34.3 | 31.8 | 37.0 |
| 16 years old | 31.7 | 28.8 | 34.7 |  | 29.5 | 27.2 | 31.9 |  | 30.9 | 28.8 | 33.1 |
| 17 years old | 31.4 | 28.6 | 34.4 |  | 28.8 | 26.1 | 31.7 |  | 28.5 | 25.5 | 31.7 |
|  |  |  |  |  |  |  |  |  |  |  |  |
| **Race** |  |  |  |  |  |  |  |  |  |  |  |
| White | 35.1 | 32.0 | 38.3 |  | 35.4 | 33.4 | 37.4 |  | 37.0 | 33.9 | 40.3 |
| Black or African American | 29.9 | 25.8 | 34.4 |  | 25.6 | 22.1 | 29.4 |  | 23.9 | 21.5 | 26.5 |
| Hispanic/Latino | 31.1 | 28.6 | 33.8 |  | 27.4 | 24.6 | 30.4 |  | 25.4 | 22.8 | 28.1 |
| All Other Races | 33.3 | 29.7 | 37.1 |  | 25.8 | 23.0 | 28.8 |  | 29.5 | 25.6 | 33.8 |
|  |  |  |  |  |  |  |  |  |  |  |  |
| **Body weight** |  |  |  |  |  |  |  |  |  |  |  |
| Normal and underweight | 35.4 | 32.6 | 38.2 |  | 34.0 | 32.1 | 36.1 |  | 34.2 | 32.0 | 36.5 |
| Overweight | 30.6 | 27.5 | 34.0 |  | 26.7 | 24.2 | 29.3 |  | 28.3 | 25.6 | 31.3 |
| Obesity | 26.6 | 23.8 | 29.6 |  | 22.6 | 20.0 | 25.4 |  | 23.5 | 21.4 | 25.8 |
|  |  |  |  |  |  |  |  |  |  |  |  |
| **Recreational screen time per day** |  |  |  |  |  |  |  |  |  |  |  |
| No more than 2 hours | 38.4 | 34.3 | 42.6 |  | 37.8 | 35.1 | 40.5 |  | 39.2 | 36.2 | 42.2 |
| More than 2 hours | 30.8 | 28.9 | 32.7 |  | 27.9 | 26.0 | 29.8 |  | 29.2 | 27.2 | 31.2 |

**Supplementary Table 8. Weighted prevalence of seven days of moderate to vigorous physical activity per week across different survey years.**

|  | **2005** | | |  | **2007** | | |  | **2009** | | |
| --- | --- | --- | --- | --- | --- | --- | --- | --- | --- | --- | --- |
|  | **%** | **95%CI** | |  | **%** | **95%CI** | |  | **%** | **95%CI** | |
| **Overall** | 18.2 | 17.0 | 19.5 |  | 17.7 | 16.6 | 18.8 |  | 18.8 | 17.7 | 20.0 |
|  |  |  |  |  |  |  |  |  |  |  |  |
| **Sex** |  |  |  |  |  |  |  |  |  |  |  |
| Female | 12.6 | 11.4 | 13.8 |  | 11.0 | 9.7 | 12.3 |  | 11.8 | 10.5 | 13.2 |
| Male | 23.9 | 22.3 | 25.5 |  | 24.4 | 22.7 | 26.0 |  | 25.4 | 23.9 | 27.0 |
|  |  |  |  |  |  |  |  |  |  |  |  |
| **Age** |  |  |  |  |  |  |  |  |  |  |  |
| 14 years old | 17.0 | 14.5 | 19.9 |  | 19.4 | 17.0 | 22.0 |  | 21.1 | 18.5 | 23.9 |
| 15 years old | 19.6 | 17.7 | 21.7 |  | 17.7 | 16.2 | 19.3 |  | 19.8 | 18.0 | 21.8 |
| 16 years old | 18.3 | 16.6 | 20.1 |  | 18.1 | 16.5 | 19.9 |  | 19.7 | 17.7 | 21.8 |
| 17 years old | 17.0 | 15.4 | 18.7 |  | 16.3 | 14.6 | 18.2 |  | 15.9 | 14.3 | 17.7 |
|  |  |  |  |  |  |  |  |  |  |  |  |
| **Race** |  |  |  |  |  |  |  |  |  |  |  |
| White | 19.3 | 17.8 | 20.9 |  | 19.3 | 17.6 | 21.1 |  | 20.4 | 19.0 | 21.8 |
| Black or African American | 16.1 | 14.1 | 18.2 |  | 16.0 | 14.5 | 17.7 |  | 18.0 | 15.9 | 20.3 |
| Hispanic/Latino | 17.1 | 14.7 | 19.8 |  | 14.9 | 13.1 | 17.0 |  | 15.6 | 14.2 | 17.1 |
| All Other Races | 15.7 | 12.4 | 19.8 |  | 14.9 | 12.0 | 18.3 |  | 17.0 | 14.4 | 20.0 |
|  |  |  |  |  |  |  |  |  |  |  |  |
| **Body weight** |  |  |  |  |  |  |  |  |  |  |  |
| Normal and underweight | 19.1 | 17.7 | 20.6 |  | 18.0 | 16.8 | 19.2 |  | 19.7 | 18.4 | 21.0 |
| Overweight | 17.6 | 15.6 | 19.7 |  | 18.8 | 16.9 | 20.8 |  | 19.5 | 17.2 | 21.9 |
| Obesity | 14.5 | 12.6 | 16.6 |  | 15.0 | 13.2 | 17.0 |  | 13.3 | 11.5 | 15.5 |
|  |  |  |  |  |  |  |  |  |  |  |  |
| **Recreational screen time per day** |  |  |  |  |  |  |  |  |  |  |  |
| No more than 2 hours | 21.6 | 19.5 | 23.9 |  | 19.5 | 17.7 | 21.4 |  | 22.4 | 20.6 | 24.2 |
| More than 2 hours | 16.6 | 15.4 | 17.9 |  | 16.8 | 15.7 | 18.1 |  | 17.1 | 15.6 | 18.7 |
|  | **2011** | | |  | **2013** | | |  | **2015** | | |
|  | **%** | **95%CI** | |  | **%** | **95%CI** | |  | **%** | **95%CI** | |
| **Overall** | 28.8 | 27.2 | 30.5 |  | 27.2 | 25.6 | 28.9 |  | 27.4 | 25.5 | 29.3 |
|  |  |  |  |  |  |  |  |  |  |  |  |
| **Sex** |  |  |  |  |  |  |  |  |  |  |  |
| Female | 19.0 | 17.3 | 20.8 |  | 17.9 | 16.3 | 19.6 |  | 18.3 | 16.8 | 19.9 |
| Male | 38.3 | 36.2 | 40.4 |  | 36.8 | 34.7 | 38.8 |  | 36.1 | 33.6 | 38.8 |
|  |  |  |  |  |  |  |  |  |  |  |  |
| **Age** |  |  |  |  |  |  |  |  |  |  |  |
| 14 years old | 31.5 | 28.1 | 35.0 |  | 30.3 | 26.9 | 34.0 |  | 25.8 | 23.4 | 28.3 |
| 15 years old | 29.8 | 27.2 | 32.4 |  | 28.5 | 26.2 | 31.0 |  | 30.5 | 27.4 | 33.9 |
| 16 years old | 29.0 | 27.0 | 31.1 |  | 27.3 | 25.4 | 29.3 |  | 26.7 | 24.5 | 28.9 |
| 17 years old | 26.4 | 24.5 | 28.4 |  | 24.5 | 22.3 | 26.9 |  | 25.2 | 22.8 | 27.8 |
|  |  |  |  |  |  |  |  |  |  |  |  |
| **Race** |  |  |  |  |  |  |  |  |  |  |  |
| White | 30.4 | 28.2 | 32.7 |  | 28.2 | 26.0 | 30.5 |  | 29.2 | 26.7 | 31.8 |
| Black or African American | 26.3 | 24.3 | 28.4 |  | 26.2 | 23.7 | 28.8 |  | 24.4 | 20.6 | 28.6 |
| Hispanic/Latino | 26.9 | 25.1 | 28.8 |  | 25.7 | 23.3 | 28.3 |  | 24.9 | 22.4 | 27.6 |
| All Other Races | 27.3 | 24.5 | 30.4 |  | 26.3 | 23.2 | 29.7 |  | 27.1 | 23.3 | 31.3 |
|  |  |  |  |  |  |  |  |  |  |  |  |
| **Body weight** |  |  |  |  |  |  |  |  |  |  |  |
| Normal and underweight | 30.7 | 28.9 | 32.6 |  | 29.2 | 27.4 | 31.0 |  | 28.8 | 26.7 | 31.0 |
| Overweight | 25.8 | 23.0 | 29.0 |  | 24.5 | 21.3 | 28.0 |  | 25.1 | 21.8 | 28.7 |
| Obesity | 22.1 | 19.5 | 25.0 |  | 20.6 | 17.6 | 23.9 |  | 22.9 | 20.2 | 25.8 |
|  |  |  |  |  |  |  |  |  |  |  |  |
| **Recreational screen time per day** |  |  |  |  |  |  |  |  |  |  |  |
| No more than 2 hours | 34.7 | 32.1 | 37.4 |  | 34.3 | 31.0 | 37.7 |  | 32.1 | 29.4 | 35.0 |
| More than 2 hours | 26.3 | 24.9 | 27.7 |  | 24.5 | 23.1 | 25.9 |  | 25.2 | 23.4 | 27.0 |
|  | **2017** | | |  | **2019** | | |  | **2021** | | |
|  | **%** | **95%CI** | |  | **%** | **95%CI** | |  | **%** | **95%CI** | |
| **Overall** | 26.5 | 24.4 | 28.8 |  | 23.6 | 22.3 | 24.9 |  | 23.8 | 22.3 | 25.3 |
|  |  |  |  |  |  |  |  |  |  |  |  |
| **Sex** |  |  |  |  |  |  |  |  |  |  |  |
| Female | 17.5 | 15.5 | 19.6 |  | 15.9 | 14.6 | 17.4 |  | 15.4 | 13.9 | 17.0 |
| Male | 35.9 | 33.7 | 38.2 |  | 31.0 | 29.0 | 33.1 |  | 31.5 | 29.5 | 33.5 |
|  |  |  |  |  |  |  |  |  |  |  |  |
| **Age** |  |  |  |  |  |  |  |  |  |  |  |
| 14 years old | 27.2 | 23.7 | 31.1 |  | 27.1 | 23.9 | 30.6 |  | 24.2 | 21.6 | 27.0 |
| 15 years old | 28.5 | 25.9 | 31.2 |  | 25.5 | 23.2 | 27.8 |  | 26.5 | 24.5 | 28.6 |
| 16 years old | 25.4 | 22.6 | 28.3 |  | 22.0 | 20.0 | 24.2 |  | 23.0 | 21.3 | 24.8 |
| 17 years old | 25.4 | 23.0 | 27.9 |  | 21.5 | 19.4 | 23.9 |  | 21.3 | 18.8 | 23.9 |
|  |  |  |  |  |  |  |  |  |  |  |  |
| **Race** |  |  |  |  |  |  |  |  |  |  |  |
| White | 27.4 | 24.6 | 30.5 |  | 26.6 | 25.1 | 28.1 |  | 28.0 | 25.9 | 30.2 |
| Black or African American | 24.5 | 20.9 | 28.6 |  | 20.5 | 17.2 | 24.2 |  | 19.3 | 17.3 | 21.6 |
| Hispanic/Latino | 26.3 | 23.8 | 29.0 |  | 21.1 | 18.5 | 24.0 |  | 18.6 | 16.1 | 21.4 |
| All Other Races | 24.9 | 21.6 | 28.5 |  | 18.9 | 16.6 | 21.3 |  | 21.6 | 18.8 | 24.7 |
|  |  |  |  |  |  |  |  |  |  |  |  |
| **Body weight** |  |  |  |  |  |  |  |  |  |  |  |
| Normal and underweight | 28.4 | 25.9 | 31.1 |  | 25.9 | 24.4 | 27.4 |  | 25.5 | 23.8 | 27.2 |
| Overweight | 24.2 | 21.5 | 27.1 |  | 20.3 | 18.0 | 22.9 |  | 22.5 | 20.2 | 24.9 |
| Obesity | 20.3 | 18.1 | 22.7 |  | 17.0 | 14.7 | 19.5 |  | 17.8 | 16.0 | 19.7 |
|  |  |  |  |  |  |  |  |  |  |  |  |
| **Recreational screen time per day** |  |  |  |  |  |  |  |  |  |  |  |
| No more than 2 hours | 30.7 | 26.8 | 34.8 |  | 29.0 | 26.7 | 31.5 |  | 30.3 | 27.8 | 33.0 |
| More than 2 hours | 24.5 | 22.7 | 26.3 |  | 21.0 | 19.6 | 22.4 |  | 21.7 | 20.4 | 23.1 |

**Supplementary Table 9. Absolute changes in prevalence of no moderate to vigorous physical activity per week across different survey years.**

| **Group** | **Year** | **Absolute changes** | **95%CI** | | ***p*** | **Changes per cycle** | **95%CI** | |
| --- | --- | --- | --- | --- | --- | --- | --- | --- |
| **Overall sample** | 2007 | -0.19% | -2.38% | 2.00% | 0.864 | -1.15% | -1.39% | -0.90% |
|  | 2009 | -1.70% | -3.78% | 0.38% | 0.110 |  |  |  |
|  | 2011 | -10.93% | -12.69% | -9.17% | 0.000 |  |  |  |
|  | 2013 | -9.92% | -11.92% | -7.92% | 0.000 |  |  |  |
|  | 2015 | -10.80% | -12.91% | -8.70% | 0.000 |  |  |  |
|  | 2017 | -9.78% | -12.06% | -7.51% | 0.000 |  |  |  |
|  | 2019 | -8.53% | -10.81% | -6.25% | 0.000 |  |  |  |
|  | 2021 | -9.17% | -11.09% | -7.24% | 0.000 |  |  |  |
| **Sex** |  |  |  |  |  |  |  |  |
| **Female** | 2007 | 0.20% | -3.12% | 3.52% | 0.906 | -1.58% | -1.91% | -1.25% |
|  | 2009 | -1.92% | -4.79% | 0.95% | 0.189 |  |  |  |
|  | 2011 | -13.99% | -16.45% | -11.54% | 0.000 |  |  |  |
|  | 2013 | -12.39% | -15.38% | -9.41% | 0.000 |  |  |  |
|  | 2015 | -14.30% | -17.11% | -11.48% | 0.000 |  |  |  |
|  | 2017 | -12.05% | -15.56% | -8.54% | 0.000 |  |  |  |
|  | 2019 | -12.48% | -15.54% | -9.41% | 0.000 |  |  |  |
|  | 2021 | -12.63% | -15.30% | -9.97% | 0.000 |  |  |  |
| **Male** | 2007 | -0.57% | -2.62% | 1.48% | 0.586 | -0.73% | -0.96% | -0.49% |
|  | 2009 | -1.43% | -3.72% | 0.86% | 0.222 |  |  |  |
|  | 2011 | -7.94% | -9.78% | -6.10% | 0.000 |  |  |  |
|  | 2013 | -7.51% | -9.36% | -5.66% | 0.000 |  |  |  |
|  | 2015 | -7.39% | -9.63% | -5.15% | 0.000 |  |  |  |
|  | 2017 | -7.59% | -9.33% | -5.84% | 0.000 |  |  |  |
|  | 2019 | -4.67% | -6.90% | -2.43% | 0.000 |  |  |  |
|  | 2021 | -5.81% | -7.70% | -3.92% | 0.000 |  |  |  |
| **Age** |  |  |  |  |  |  |  |  |
| **14 years old** | 2007 | 1.43% | -2.32% | 5.18% | 0.454 | -1.15% | -1.54% | -0.76% |
|  | 2009 | 0.88% | -2.62% | 4.39% | 0.620 |  |  |  |
|  | 2011 | -8.90% | -12.46% | -5.35% | 0.000 |  |  |  |
|  | 2013 | -9.66% | -13.12% | -6.21% | 0.000 |  |  |  |
|  | 2015 | -10.90% | -14.27% | -7.54% | 0.000 |  |  |  |
|  | 2017 | -10.47% | -13.40% | -7.53% | 0.000 |  |  |  |
|  | 2019 | -5.51% | -9.75% | -1.28% | 0.011 |  |  |  |
|  | 2021 | -9.23% | -12.35% | -6.11% | 0.000 |  |  |  |
| **15 years old** | 2007 | -1.60% | -4.89% | 1.70% | 0.342 | -1.17% | -1.55% | -0.79% |
|  | 2009 | -0.25% | -3.73% | 3.23% | 0.887 |  |  |  |
|  | 2011 | -10.79% | -13.67% | -7.92% | 0.000 |  |  |  |
|  | 2013 | -11.01% | -13.96% | -8.06% | 0.000 |  |  |  |
|  | 2015 | -10.95% | -14.03% | -7.87% | 0.000 |  |  |  |
|  | 2017 | -10.01% | -12.90% | -7.12% | 0.000 |  |  |  |
|  | 2019 | -10.21% | -13.36% | -7.05% | 0.000 |  |  |  |
|  | 2021 | -9.35% | -12.41% | -6.29% | 0.000 |  |  |  |
| **16 years old** | 2007 | 1.38% | -1.82% | 4.59% | 0.396 | -1.01% | -1.32% | -0.71% |
|  | 2009 | -1.92% | -4.60% | 0.77% | 0.161 |  |  |  |
|  | 2011 | -9.75% | -12.13% | -7.36% | 0.000 |  |  |  |
|  | 2013 | -9.59% | -12.22% | -6.96% | 0.000 |  |  |  |
|  | 2015 | -9.24% | -11.98% | -6.49% | 0.000 |  |  |  |
|  | 2017 | -9.48% | -12.53% | -6.43% | 0.000 |  |  |  |
|  | 2019 | -7.32% | -10.16% | -4.49% | 0.000 |  |  |  |
|  | 2021 | -8.09% | -10.53% | -5.65% | 0.000 |  |  |  |
| **17 years old** | 2007 | -1.14% | -3.97% | 1.70% | 0.431 | -1.23% | -1.59% | -0.86% |
|  | 2009 | -4.13% | -7.11% | -1.15% | 0.007 |  |  |  |
|  | 2011 | -13.39% | -15.91% | -10.87% | 0.000 |  |  |  |
|  | 2013 | -9.43% | -12.55% | -6.31% | 0.000 |  |  |  |
|  | 2015 | -12.36% | -15.40% | -9.33% | 0.000 |  |  |  |
|  | 2017 | -9.60% | -13.18% | -6.02% | 0.000 |  |  |  |
|  | 2019 | -9.61% | -12.80% | -6.41% | 0.000 |  |  |  |
|  | 2021 | -9.81% | -12.75% | -6.88% | 0.000 |  |  |  |
| **Race** |  |  |  |  |  |  |  |  |
| **White** | 2007 | -0.22% | -3.02% | 2.58% | 0.877 | -1.23% | -1.52% | -0.93% |
|  | 2009 | -2.20% | -4.58% | 0.17% | 0.069 |  |  |  |
|  | 2011 | -10.95% | -12.99% | -8.91% | 0.000 |  |  |  |
|  | 2013 | -9.40% | -11.83% | -6.96% | 0.000 |  |  |  |
|  | 2015 | -10.70% | -13.31% | -8.09% | 0.000 |  |  |  |
|  | 2017 | -9.00% | -11.91% | -6.10% | 0.000 |  |  |  |
|  | 2019 | -9.45% | -11.77% | -7.12% | 0.000 |  |  |  |
|  | 2021 | -9.82% | -12.19% | -7.46% | 0.000 |  |  |  |
| **Black or African American** | 2007 | -1.18% | -5.39% | 3.03% | 0.582 | -1.05% | -1.61% | -0.48% |
|  | 2009 | 0.04% | -4.68% | 4.76% | 0.987 |  |  |  |
|  | 2011 | -12.85% | -17.00% | -8.71% | 0.000 |  |  |  |
|  | 2013 | -11.89% | -15.62% | -8.15% | 0.000 |  |  |  |
|  | 2015 | -11.41% | -16.15% | -6.68% | 0.000 |  |  |  |
|  | 2017 | -11.72% | -15.78% | -7.66% | 0.000 |  |  |  |
|  | 2019 | -5.36% | -10.46% | -0.27% | 0.039 |  |  |  |
|  | 2021 | -8.37% | -12.87% | -3.86% | 0.000 |  |  |  |
| **Hispanic/Latino** | 2007 | 0.16% | -4.05% | 4.37% | 0.940 | -0.86% | -1.36% | -0.37% |
|  | 2009 | -2.16% | -5.80% | 1.49% | 0.245 |  |  |  |
|  | 2011 | -10.00% | -13.75% | -6.25% | 0.000 |  |  |  |
|  | 2013 | -10.10% | -14.05% | -6.16% | 0.000 |  |  |  |
|  | 2015 | -10.58% | -14.77% | -6.40% | 0.000 |  |  |  |
|  | 2017 | -10.30% | -14.11% | -6.49% | 0.000 |  |  |  |
|  | 2019 | -7.54% | -12.08% | -3.01% | 0.001 |  |  |  |
|  | 2021 | -6.91% | -10.87% | -2.95% | 0.001 |  |  |  |
| **All other races** | 2007 | 1.06% | -4.74% | 6.86% | 0.719 | -1.08% | -1.65% | -0.51% |
|  | 2009 | 0.86% | -4.54% | 6.25% | 0.755 |  |  |  |
|  | 2011 | -8.12% | -13.23% | -3.02% | 0.002 |  |  |  |
|  | 2013 | -7.78% | -12.56% | -3.00% | 0.001 |  |  |  |
|  | 2015 | -8.87% | -14.15% | -3.59% | 0.001 |  |  |  |
|  | 2017 | -7.73% | -12.58% | -2.89% | 0.002 |  |  |  |
|  | 2019 | -7.04% | -11.97% | -2.12% | 0.005 |  |  |  |
|  | 2021 | -8.65% | -13.23% | -4.07% | 0.000 |  |  |  |

CI: confidence interval.

**Supplementary Table 9. Continued.**

| **Group** | **Year** | **Absolute changes** | **95%CI** | | ***p*** | **Changes per cycle** | **95%CI** | |
| --- | --- | --- | --- | --- | --- | --- | --- | --- |
| **Body weight** |  |  |  |  |  |  |  |  |
| **Normal and underweight** | 2007 | -0.44% | -3.01% | 2.13% | 0.738 | -1.21% | -1.48% | -0.94% |
|  | 2009 | -2.12% | -4.37% | 0.12% | 0.063 |  |  |  |
|  | 2011 | -10.80% | -12.75% | -8.85% | 0.000 |  |  |  |
|  | 2013 | -9.79% | -11.85% | -7.73% | 0.000 |  |  |  |
|  | 2015 | -10.78% | -13.10% | -8.46% | 0.000 |  |  |  |
|  | 2017 | -9.95% | -12.37% | -7.53% | 0.000 |  |  |  |
|  | 2019 | -8.80% | -11.21% | -6.39% | 0.000 |  |  |  |
|  | 2021 | -9.67% | -11.81% | -7.52% | 0.000 |  |  |  |
| **Overweight** | 2007 | -0.09% | -4.20% | 4.03% | 0.967 | -1.14% | -1.56% | -0.73% |
|  | 2009 | -1.85% | -5.55% | 1.85% | 0.327 |  |  |  |
|  | 2011 | -13.49% | -16.54% | -10.43% | 0.000 |  |  |  |
|  | 2013 | -11.66% | -15.19% | -8.14% | 0.000 |  |  |  |
|  | 2015 | -10.95% | -14.41% | -7.49% | 0.000 |  |  |  |
|  | 2017 | -10.55% | -14.22% | -6.89% | 0.000 |  |  |  |
|  | 2019 | -8.61% | -12.52% | -4.70% | 0.000 |  |  |  |
|  | 2021 | -9.15% | -12.49% | -5.82% | 0.000 |  |  |  |
| **Obesity** | 2007 | 0.87% | -2.73% | 4.48% | 0.634 | -0.87% | -1.31% | -0.44% |
|  | 2009 | 0.62% | -3.64% | 4.89% | 0.774 |  |  |  |
|  | 2011 | -8.77% | -12.96% | -4.57% | 0.000 |  |  |  |
|  | 2013 | -8.56% | -12.54% | -4.57% | 0.000 |  |  |  |
|  | 2015 | -10.76% | -14.43% | -7.09% | 0.000 |  |  |  |
|  | 2017 | -8.19% | -11.95% | -4.42% | 0.000 |  |  |  |
|  | 2019 | -7.16% | -11.41% | -2.91% | 0.001 |  |  |  |
|  | 2021 | -6.98% | -10.45% | -3.51% | 0.000 |  |  |  |
| **Screen time per day** |  |  |  |  |  |  |  |  |
| **No more than 2 hours** | 2007 | -0.35% | -3.25% | 2.55% | 0.812 | -0.59% | -0.95% | -0.23% |
|  | 2009 | -2.07% | -4.77% | 0.63% | 0.133 |  |  |  |
|  | 2011 | -10.40% | -12.98% | -7.82% | 0.000 |  |  |  |
|  | 2013 | -9.25% | -12.27% | -6.22% | 0.000 |  |  |  |
|  | 2015 | -8.65% | -12.04% | -5.26% | 0.000 |  |  |  |
|  | 2017 | -7.25% | -10.71% | -3.79% | 0.000 |  |  |  |
|  | 2019 | -5.60% | -9.15% | -2.05% | 0.002 |  |  |  |
|  | 2021 | -4.76% | -7.63% | -1.88% | 0.001 |  |  |  |
| **More than 2 hours** | 2007 | -0.10% | -2.55% | 2.34% | 0.934 | -1.34% | -1.60% | -1.08% |
|  | 2009 | -1.48% | -3.76% | 0.81% | 0.204 |  |  |  |
|  | 2011 | -11.19% | -13.04% | -9.34% | 0.000 |  |  |  |
|  | 2013 | -10.30% | -12.62% | -7.97% | 0.000 |  |  |  |
|  | 2015 | -11.78% | -13.81% | -9.75% | 0.000 |  |  |  |
|  | 2017 | -10.98% | -13.04% | -8.91% | 0.000 |  |  |  |
|  | 2019 | -9.89% | -12.10% | -7.68% | 0.000 |  |  |  |
|  | 2021 | -10.71% | -12.79% | -8.64% | 0.000 |  |  |  |

CI: confidence interval.

**Supplementary Table 10. Interaction effects of survey year and covariates on no moderate-to-vigorous physical activity.**

| **No moderate to vigorous physical activity** |  | **95%CI** | | ***p*** | **F statistic** | ***p*** |
| --- | --- | --- | --- | --- | --- | --- |
| **Survey year × sex (reference = female)** |  |  |  |  |  |  |
| 2007 × Male | -0.05 | -0.23 | 0.12 | 0.569 | F (8, 403) = 3.00 | 0.0028 |
| 2009 × Male | -0.01 | -0.19 | 0.17 | 0.909 |  |  |
| 2011 × Male | 0.11 | -0.06 | 0.28 | 0.198 |  |  |
| 2013 × Male | 0.05 | -0.15 | 0.24 | 0.641 |  |  |
| 2015 × Male | 0.18 | -0.02 | 0.39 | 0.081 |  |  |
| 2017 × Male | 0.01 | -0.18 | 0.21 | 0.917 |  |  |
| 2019 × Male | 0.32 | 0.14 | 0.50 | 0.001 |  |  |
| 2021 × Male | 0.23 | 0.07 | 0.39 | 0.006 |  |  |
| **Survey year × age (reference = 14 years)** |  |  |  |  |  |  |
| 2007 × 15 years old | -0.20 | -0.44 | 0.05 | 0.111 | F (24, 387) = 2.49 | 0.0002 |
| 2007 × 16 years old | -0.01 | -0.25 | 0.24 | 0.957 |  |  |
| 2007 × 17 years old | -0.14 | -0.37 | 0.09 | 0.224 |  |  |
| 2009 × 15 years old | -0.08 | -0.32 | 0.17 | 0.530 |  |  |
| 2009 × 16 years old | -0.17 | -0.38 | 0.05 | 0.126 |  |  |
| 2009 × 17 years old | -0.28 | -0.51 | -0.04 | 0.020 |  |  |
| 2011 × 15 years old | -0.11 | -0.44 | 0.22 | 0.505 |  |  |
| 2011 × 16 years old | 0.02 | -0.30 | 0.35 | 0.890 |  |  |
| 2011 × 17 years old | -0.16 | -0.48 | 0.17 | 0.340 |  |  |
| 2013 × 15 years old | -0.04 | -0.36 | 0.28 | 0.791 |  |  |
| 2013 × 16 years old | 0.11 | -0.21 | 0.44 | 0.491 |  |  |
| 2013 × 17 years old | 0.21 | -0.13 | 0.55 | 0.230 |  |  |
| 2015 × 15 years old | 0.09 | -0.20 | 0.37 | 0.553 |  |  |
| 2015 × 16 years old | 0.27 | -0.01 | 0.56 | 0.056 |  |  |
| 2015 × 17 years old | 0.13 | -0.19 | 0.45 | 0.418 |  |  |
| 2017 × 15 years old | 0.13 | -0.14 | 0.40 | 0.353 |  |  |
| 2017 × 16 years old | 0.21 | -0.06 | 0.49 | 0.126 |  |  |
| 2017 × 17 years old | 0.29 | 0.02 | 0.55 | 0.034 |  |  |
| 2019 × 15 years old | -0.35 | -0.63 | -0.06 | 0.017 |  |  |
| 2019 × 16 years old | -0.07 | -0.39 | 0.25 | 0.657 |  |  |
| 2019 × 17 years old | -0.17 | -0.46 | 0.11 | 0.239 |  |  |
| 2021 × 15 years old | 0.04 | -0.20 | 0.28 | 0.714 |  |  |
| 2021 × 16 years old | 0.18 | -0.07 | 0.43 | 0.154 |  |  |
| 2021 × 17 years old | 0.13 | -0.12 | 0.37 | 0.317 |  |  |
| **Survey year × race (reference = White)** |  |  |  |  |  |  |
| 2007 × Black or African American | -0.04 | -0.30 | 0.21 | 0.738 | F (24, 387) = 1.02 | 0.4327 |
| 2007 × Hispanic/Latino | 0.02 | -0.24 | 0.29 | 0.862 |  |  |
| 2007 × All Other Races | 0.07 | -0.30 | 0.44 | 0.699 |  |  |
| 2009 × Black or African American | 0.14 | -0.12 | 0.40 | 0.284 |  |  |
| 2009 × Hispanic/Latino | 0.02 | -0.22 | 0.26 | 0.862 |  |  |
| 2009 × All Other Races | 0.18 | -0.14 | 0.51 | 0.271 |  |  |
| 2011 × Black or African American | 0.12 | -0.15 | 0.38 | 0.394 |  |  |
| 2011 × Hispanic/Latino | 0.21 | -0.06 | 0.48 | 0.131 |  |  |
| 2011 × All Other Races | 0.32 | -0.03 | 0.67 | 0.070 |  |  |
| 2013 × Black or African American | 0.03 | -0.24 | 0.29 | 0.829 |  |  |
| 2013 × Hispanic/Latino | 0.05 | -0.23 | 0.33 | 0.742 |  |  |
| 2013 × All Other Races | 0.19 | -0.15 | 0.54 | 0.267 |  |  |
| 2015 × Black or African American | 0.19 | -0.16 | 0.53 | 0.292 |  |  |
| 2015 × Hispanic/Latino | 0.14 | -0.19 | 0.46 | 0.411 |  |  |
| 2015 × All Other Races | 0.24 | -0.19 | 0.67 | 0.277 |  |  |
| 2017 × Black or African American | 0.00 | -0.27 | 0.28 | 0.981 |  |  |
| 2017 × Hispanic/Latino | 0.00 | -0.32 | 0.31 | 0.987 |  |  |
| 2017 × All Other Races | 0.16 | -0.19 | 0.51 | 0.361 |  |  |
| 2019 × Black or African American | 0.42 | 0.10 | 0.73 | 0.010 |  |  |
| 2019 × Hispanic/Latino | 0.24 | -0.07 | 0.55 | 0.133 |  |  |
| 2019 × All Other Races | 0.25 | -0.07 | 0.58 | 0.127 |  |  |
| 2021 × Black or African American | 0.29 | -0.05 | 0.63 | 0.099 |  |  |
| 2021 × Hispanic/Latino | 0.32 | 0.05 | 0.59 | 0.021 |  |  |
| 2021 × All Other Races | 0.17 | -0.14 | 0.48 | 0.291 |  |  |
| **Survey year × body weight (reference = Normal and underweight)** |  |  |  |  |  |  |
| 2007 × Overweight | 0.03 | -0.20 | 0.26 | 0.796 | F (16, 395) = 1.00 | 0.4526 |
| 2007 × Obesity | 0.08 | -0.14 | 0.30 | 0.476 |  |  |
| 2009 × Overweight | 0.04 | -0.16 | 0.23 | 0.716 |  |  |
| 2009 × Obesity | 0.17 | -0.07 | 0.40 | 0.161 |  |  |
| 2011 × Overweight | -0.18 | -0.41 | 0.05 | 0.130 |  |  |
| 2011 × Obesity | 0.22 | -0.06 | 0.49 | 0.118 |  |  |
| 2013 × Overweight | -0.11 | -0.34 | 0.13 | 0.374 |  |  |
| 2013 × Obesity | 0.13 | -0.09 | 0.35 | 0.246 |  |  |
| 2015 × Overweight | 0.04 | -0.20 | 0.28 | 0.730 |  |  |
| 2015 × Obesity | 0.07 | -0.17 | 0.30 | 0.584 |  |  |
| 2017 × Overweight | 0.01 | -0.20 | 0.22 | 0.935 |  |  |
| 2017 × Obesity | 0.19 | -0.03 | 0.42 | 0.093 |  |  |
| 2019 × Overweight | 0.06 | -0.20 | 0.31 | 0.661 |  |  |
| 2019 × Obesity | 0.15 | -0.09 | 0.40 | 0.215 |  |  |
| 2021 × Overweight | 0.09 | -0.12 | 0.29 | 0.404 |  |  |
| 2021 × Obesity | 0.25 | 0.03 | 0.46 | 0.024 |  |  |
| **Survey year × recreational screen time (reference = no more than 2 hours)** |  |  |  |  |  |  |
| 2007 **×** more than 2 hours per day | 0.01 | -0.17 | 0.19 | 0.925 | F (8, 403) = 2.96 | 0.0032 |
| 2009 × more than 2 hours per day | 0.06 | -0.11 | 0.22 | 0.500 |  |  |
| 2011 × more than 2 hours per day | 0.08 | -0.12 | 0.28 | 0.419 |  |  |
| 2013 × more than 2 hours per day | 0.04 | -0.25 | 0.33 | 0.774 |  |  |
| 2015 × more than 2 hours per day | -0.12 | -0.36 | 0.13 | 0.344 |  |  |
| 2017 × more than 2 hours per day | -0.18 | -0.38 | 0.03 | 0.102 |  |  |
| 2019 × more than 2 hours per day | -0.21 | -0.44 | 0.01 | 0.063 |  |  |
| 2021 × more than 2 hours per day | -0.33 | -0.52 | -0.13 | 0.001 |  |  |

CI: confidence interval


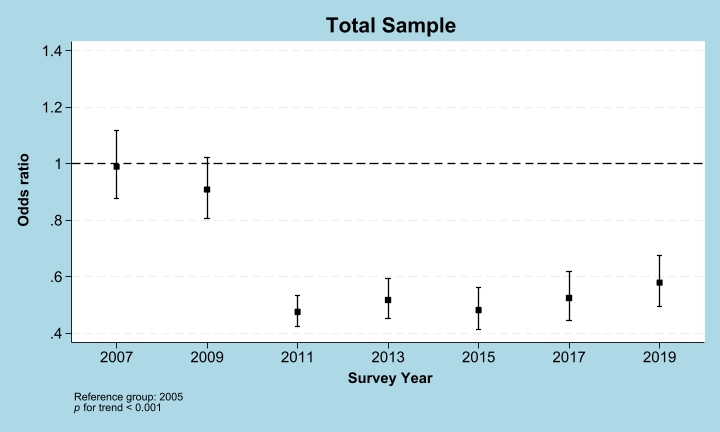


Supplementary Figure 1. Odds ratio for no moderate to vigorous physical activity across different survey years in the total sample.

The analysis for was adjusted for sex, age, race, body weight and screen time.


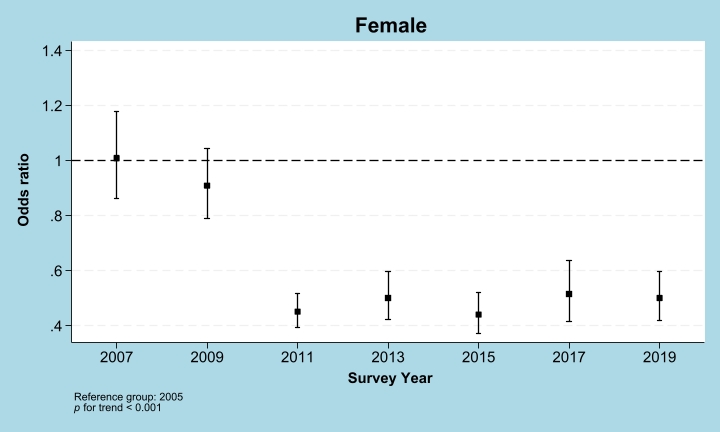


Supplementary Figure 2. Odds ratio for no moderate to vigorous physical activity across different survey years in those of females.

The analysis for was adjusted for age, race, body weight and screen time.


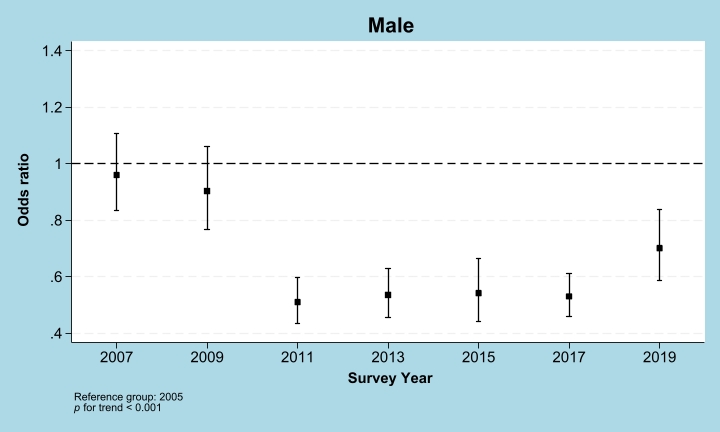


Supplementary Figure 3. Odds ratio for no moderate to vigorous physical activity across different survey years in those of males.

The analysis for was adjusted for age, race, body weight and screen time.


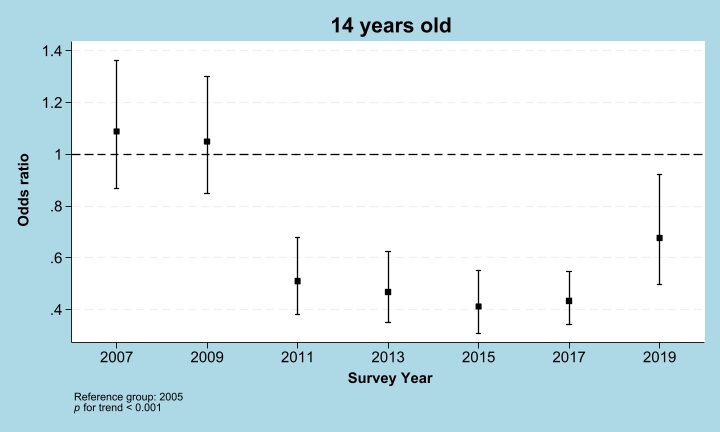


Supplementary Figure 4. Odds ratio for no moderate to vigorous physical activity across different survey years in those of 14 years old.

The analysis for was adjusted for sex, race, body weight and screen time.


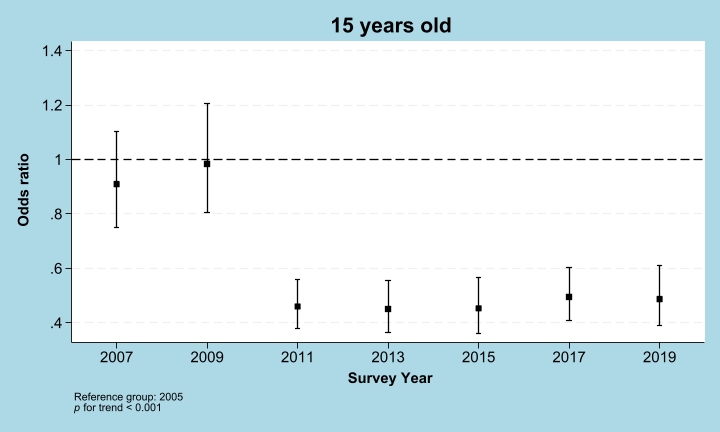


Supplementary Figure 5. Odds ratio for no moderate to vigorous physical activity across different survey years in those of 15 years old.

The analysis for was adjusted for sex, race, body weight and screen time.


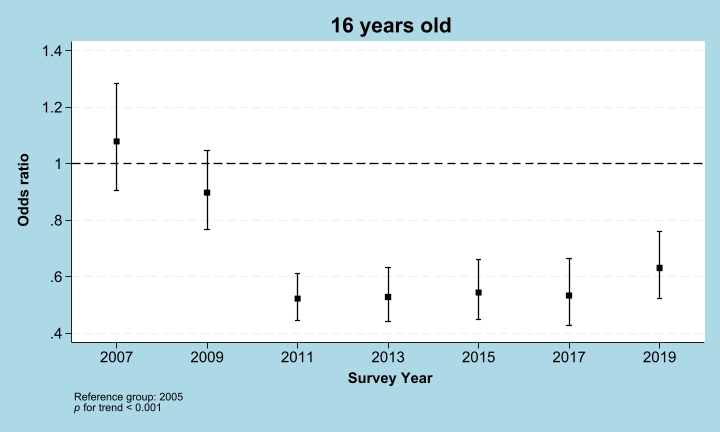


Supplementary Figure 6. Odds ratio for no moderate to vigorous physical activity across different survey years in those of 16 years old.

The analysis for was adjusted for sex, race, body weight and screen time.


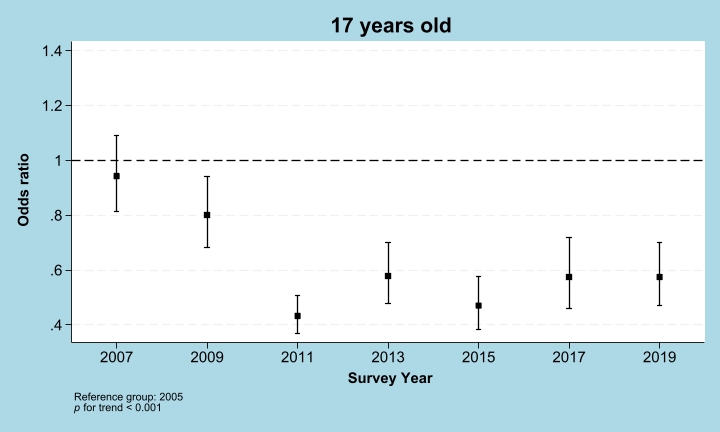


Supplementary Figure 7. Odds ratio for no moderate to vigorous physical activity across different survey years in those of 17 years old.

The analysis for was adjusted for sex, race, body weight and screen time.


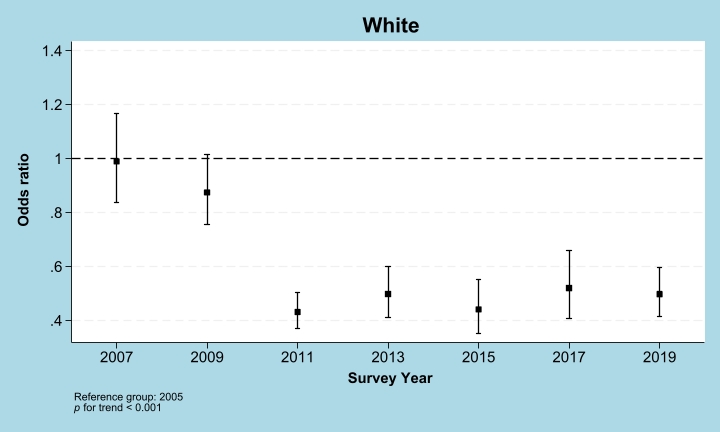


Supplementary Figure 8. Odds ratio for no moderate to vigorous physical activity across different survey years in those of White.

The analysis for was adjusted for age, sex, body weight and screen time.


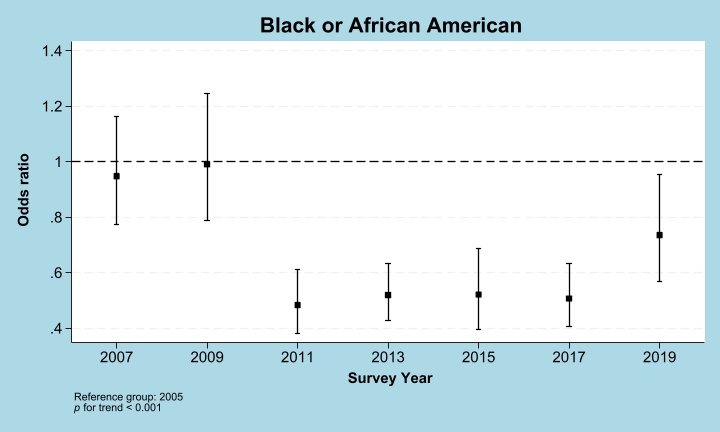


Supplementary Figure 9. Odds ratio for no moderate to vigorous physical activity across different survey years in those of Black or African American.

The analysis for was adjusted for age, sex, body weight and screen time.


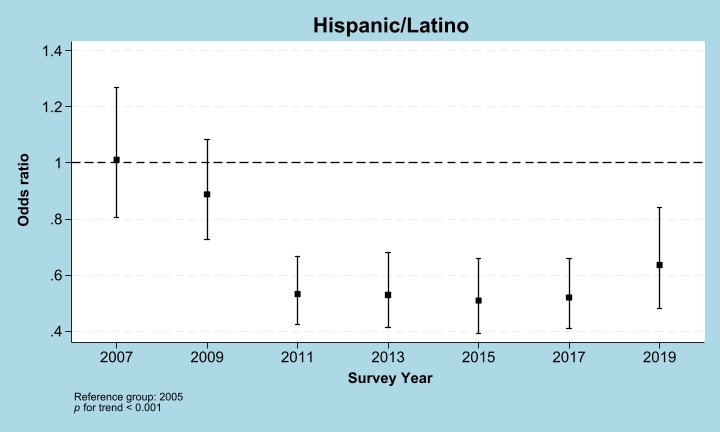


Supplementary Figure 10. Odds ratio for no moderate to vigorous physical activity across different survey years in those of Hispanic/Latino.

The analysis for was adjusted for age, sex, body weight and screen time.


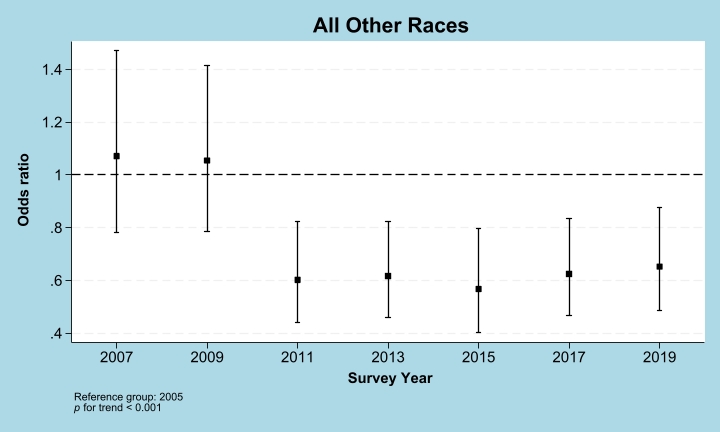


Supplementary Figure 11. Odds ratio for no moderate to vigorous physical activity across different survey years in those of all other races.

The analysis for was adjusted for age, sex, body weight and screen time.


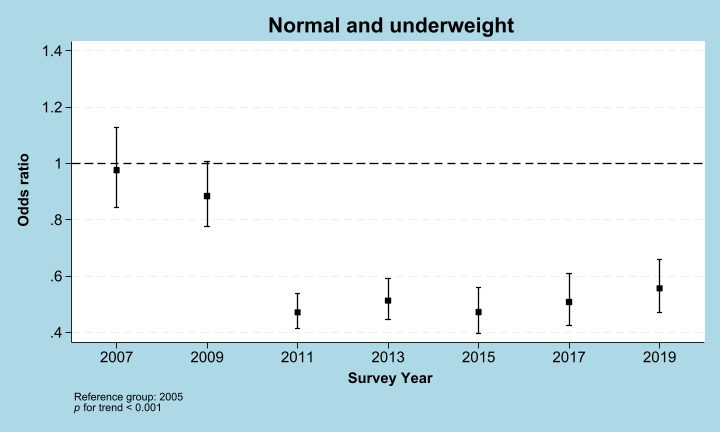


Supplementary Figure 12. Odds ratio for no moderate to vigorous physical activity across different survey years in those who are normal and underweight.

The analysis for was adjusted for age, sex, race and screen time.


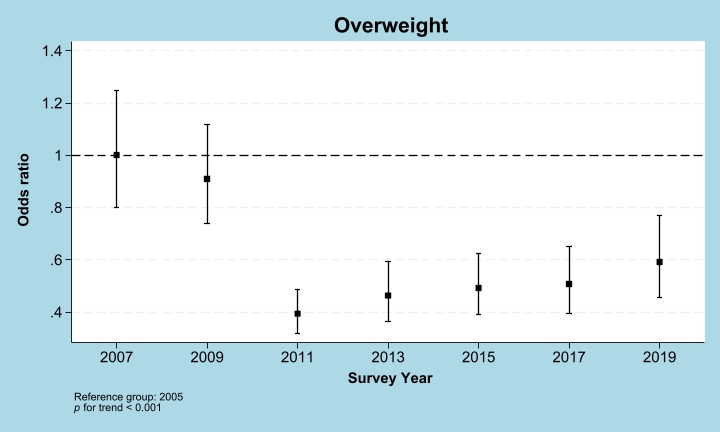


Supplementary Figure 13. Odds ratio for no moderate to vigorous physical activity across different survey years in those with overweight.

The analysis for was adjusted for age, sex, race and screen time.


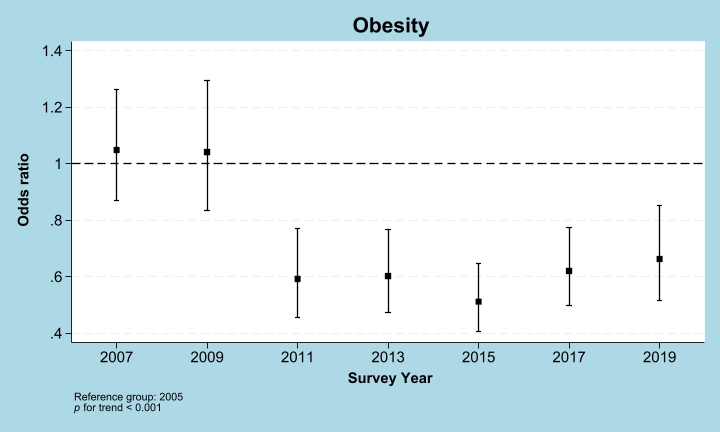


Supplementary Figure 14. Odds ratio for no moderate to vigorous physical activity across different survey years in those with obesity.

The analysis for was adjusted for age, sex, race and screen time.


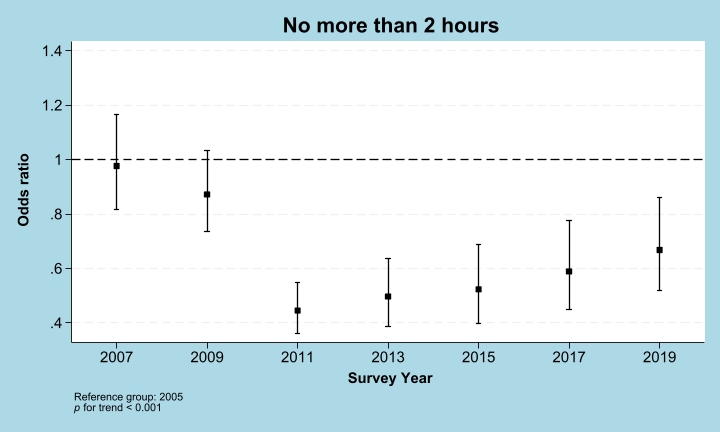


Supplementary Figure 15. Odds ratio for no moderate to vigorous physical activity across different survey years in those with no more than 2 hours of screen time per day.

The analysis for was adjusted for age, sex, race and body weight.


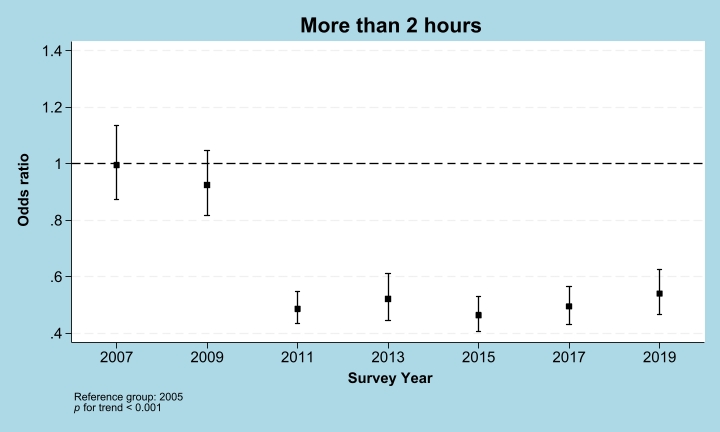


Supplementary Figure 16. Odds ratio for no moderate to vigorous physical activity across different survey years in those with more than 2 hours of screen time per day.

The analysis for was adjusted for age, sex, race and body weight.
